# Charge-tagged ligands: useful tools for immobilising complexes and detecting reaction species during catalysis

**DOI:** 10.1039/c4sc02151g

**Published:** 2014-08-06

**Authors:** Jones Limberger, Bárbara C. Leal, Adriano L. Monteiro, Jairton Dupont

**Affiliations:** a Laboratory of Molecular Catalysis , Institute of Chemistry – UFRGS , Av. Bento Gonçalves 9500, 91501-970, CP 15003 , Porto Alegre , RS , Brazil . Fax: +55(51)33087304; b School of Chemistry , University of Nottingham , University Park , Nottingham , NG7 2RD , UK . Email: jairton.dupont@ufrgs.br

## Abstract

In recent years, charge-tagged ligands (CTLs) have become valuable tools in organometallic catalysis. Insertion of an ionic side chain into the molecular skeleton of a known ligand has become a useful protocol for anchoring ligands, and consequently catalysts, in polar and ionic liquid phases. In addition, the insertion of a cationic moiety into a ligand is a powerful tool that can be used to detect reaction intermediates in organometallic catalysis through electrospray ionisation mass spectrometry (ESI-MS) experiments. The insertion of an ionic tag ensures the charge in the intermediates independently of the ESI-MS. For this reason, these ligands have been used as ionic probes in mechanistic studies for several catalytic reactions. Here, we summarise selected examples on the use of CTLs as immobilising agents in organometallic catalysis and as probes for studying mechanisms through ESI-MS.

## Abstract 2

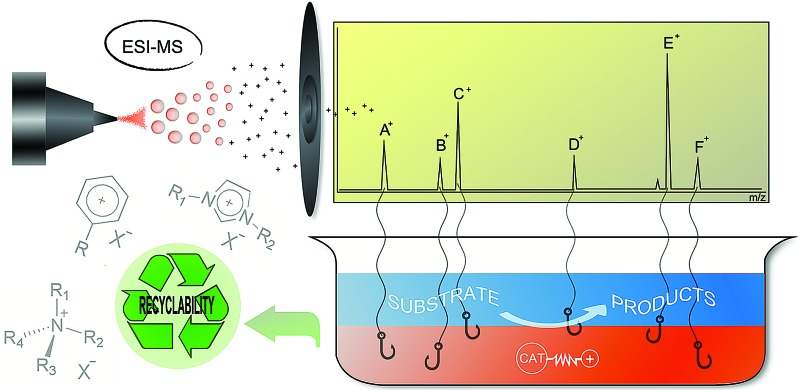
A critical overview is presented on the use of charged tagged ligands (CTLs) as immobilising agents in organometallic catalysis and as probes for studying mechanisms through electrospray ionisation mass spectrometry (ESI-MS) based on the most recent literature.

## Introduction

Undoubtedly, the choice of an appropriate solvent is vital for any organic transformation.^1^ The reaction media can dramatically affect the yield and selectivity of a reaction, owing to several factors including the reagents, the solubility of the products and catalysts as well as charge stabilisation. The importance of this issue is increased in homogeneous biphasic catalysis. Indeed, an incorrect solvent pairing can lead to low conversions, poor product extraction, no phase separation and catalyst lixiviation.^2^ Historically, one of the best strategies to avoid lixiviation is to insert organic moieties into the ligand structure with the same features for anchoring the media,^3^ for example fluorinated ligands in organofluorinated solvents,^4,5^ polyethyleneglycol (PEG)-substituted ligands in PEG media^6–8^ and charged ligands in highly polar solvents.^9–11^

The use of charged ligands has attracted special attention, because water can be used as an anchoring medium, thereby combining sustainability and efficiency.^11,12^ The first successful protocol was the application of monosulphonated phosphines in rhodium-catalysed propene hydroformylation.^13–15^

With the emergence of ionic liquids (ILs), the insertion of an ion-tagged side chain into the molecular skeleton of a known ligand became a useful protocol for anchoring ligands, and consequently catalysts, in an IL phase.^16^ These ligands are referred to as ionophilic ligands or charge-tagged ligands (CTLs). The ionic modification confers a particular solubility profile that makes catalyst/product recovery possible, and often improves the activity of the catalytic species compared to the parent tag-free analogue.^17^ Moreover, new selective processes can be envisaged by changing the solubility and diffusion of substrates and products in the active phase of a reaction.^18^

**Scheme 1:**
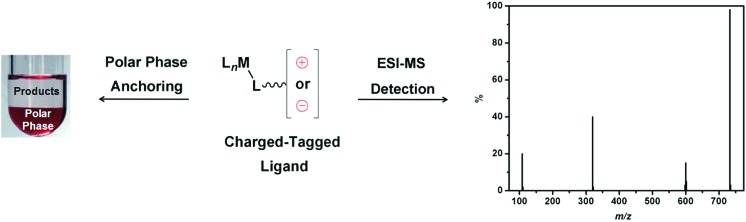
CTLs for polar phase anchoring and ESI-MS detection.

In addition to the change in catalyst partition, the insertion of a cationic moiety into various reagents represents a powerful tool that can be used to detect reaction intermediates in organometallic catalysis through electrospray ionisation mass spectrometry (ESI-MS) experiments.^19^ Taking into account the fact that ESI constitutes a mild ionisation technique, some neutral intermediates may not be ionised and, therefore, are not detected by MS.^19^ To overcome the detection problem of these “ESI-MS-blind intermediates”, CTLs have been used.^20,21^ The insertion of an ionic tag ensures that the charge of an intermediate is not caused by the ESI. For this reason, these ligands have been used as ionic probes in mechanistic studies for several catalytic reactions. Scheme 1 illustrates the application of CTLs for both polar phase anchoring and ESI-MS detection.

There are extensive reviews covering both ESI-MS detection and the anchoring processes; a review of catalysts with ionic tags and their use in ILs was published in 2008.^16^ In the same year, a perspective review of using charged ligands for catalyst immobilisation and analysis appeared.^21^ In addition, it was shown that the use of a proper ionic tag can improve the performance of catalytic systems.^22^ Herein, we summarise examples from our laboratory and, where appropriate, recent literature studies on the use of CTLs as immobilising agents in organometallic catalysis and as probes for studying mechanisms through ESI-MS.

### CTLs as immobilising agents for organometallic catalyst precursors

#### Olefin metathesis reactions

**Scheme 2:**
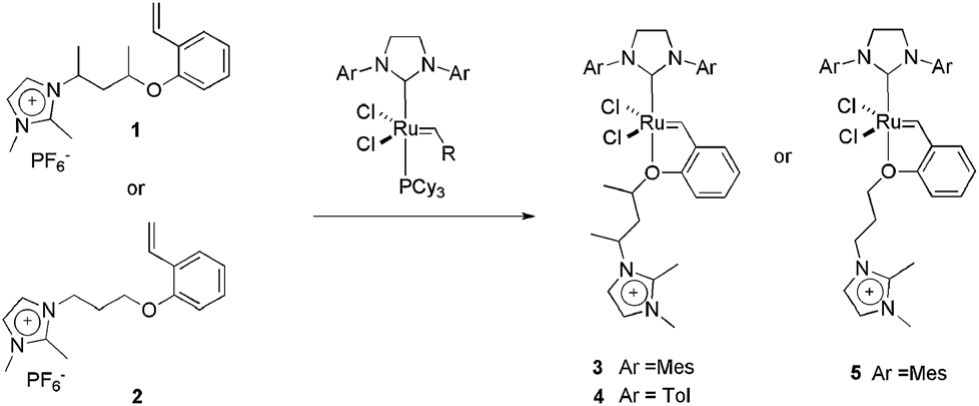
Structure of CTLs **1** and **2** and the synthesis of charge-tagged Hoveyda-type catalysts **3**, **4** and **5**.

Our group has dedicated the recent years to synthesising imidazolium-based metathesis CTLs. We have used these ligands to immobilise homogeneous catalysts in ILs. CTLs **1** and **2**, for example, were easily obtained using three-step routes. These ligands were reacted with a second-generation Grubbs catalyst to produce the ionophilic Hoveyda-type complexes **3**, **4** and **5** (Scheme 2).^23^ These complexes were successfully applied to ring-closing metathesis (RCM) reactions. Initially, it was observed that a judicious choice of anchoring IL is crucial for both activity and reusability. With the optimised conditions (**3**, BMI·PF_6_ (BMI = 1-butyl-3-methylimidazolium), 45 °C, toluene co-solvent), turnover frequencies (TOFs) of up to 343 000 h^–1^ were obtained in the 1,7-octadiene RCM reaction, even at ppm concentration levels. The reusability of the system was tested in the RCM of diallyldiethylmalonate, and seven cycles could be performed without an apparent decrease in activity. Another noteworthy finding is the fact that a high degree of substitution at the carbon attached to the oxygen in the ether portion of the CTL is beneficial to the catalytic activity.

We also successfully used CTL **3** in a tandem isomerisation–metathesis reaction of *trans*-3-hexene, affording heavier linear olefins.^18^ The reaction was performed in a biphasic system composed of BMI·PF_6_ and toluene. In this way, the metathesis catalyst, **1**, was anchored in the IL phase and the isomerisation catalyst, RuHClCO(PPh_3_)_3_, was kept in the organic phase. The use of this biphasic system is essential for keeping a superior olefin concentration in the toluene phase and, consequently, ensuring the correct isomerisation–metathesis rate. In this way, 80% of the initial *trans*-3-hexene was converted to an olefinic C4–C17 mixture, with 49% of the linear olefins being higher than C6.

**Fig. 1:**
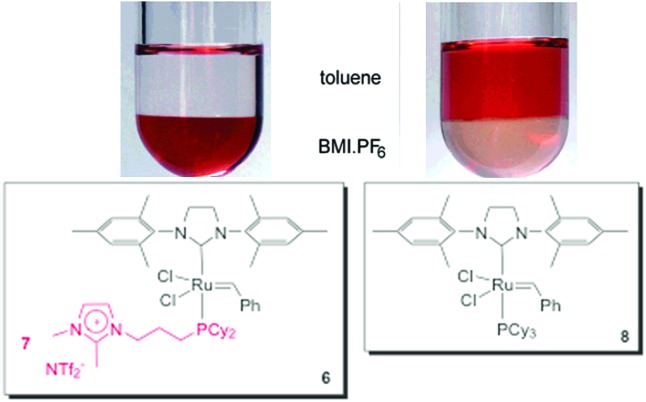
Comparison of the distribution of **6** and **8** in a biphasic system composed of BMI·PF_6_ and toluene. Adapted from ref. 24. Copyright (2008) American Chemical Society.

As well as Hoveyda-type catalysts, we have also produced the charge-tagged Grubbs-type catalyst **6**. In this case, the ionic tag was provided by the ionophilic phosphine **7**. The synthesis of this CTL is easy, and could be achieved through a one-step radical chain addition of secondary phosphines to allyl imidazolium salts.^24^ The charge-tagged ruthenium complex **6** was applied to the RCM of 1,7-octadiene. When this reaction was performed in BMI·PF_6_–toluene, it afforded cyclohexene in a 98% yield with only 0.25 mol% of the catalyst. Moreover, this system could be recycled eight times without loss of activity. This reusability was superior to that of the second-generation Grubbs catalyst, which exhibited a pronounced decrease in activity in the second cycle. Atomic absorption analysis of the toluene phase after each cycle, when using complex **6**, revealed a ruthenium content below the detection limits of the technique (<2 ppm, *i.e.*, less than 1.5% of the initial Ru content). Unfortunately, low conversions were observed when the system was applied to the RCM of highly substituted dienes. Fig. 1 illustrates the distribution of **6** and **8** (Grubbs catalyst) over the IL and toluene phases. Note that the toluene phase is uncoloured when using complex **6**.

**Scheme 3:**
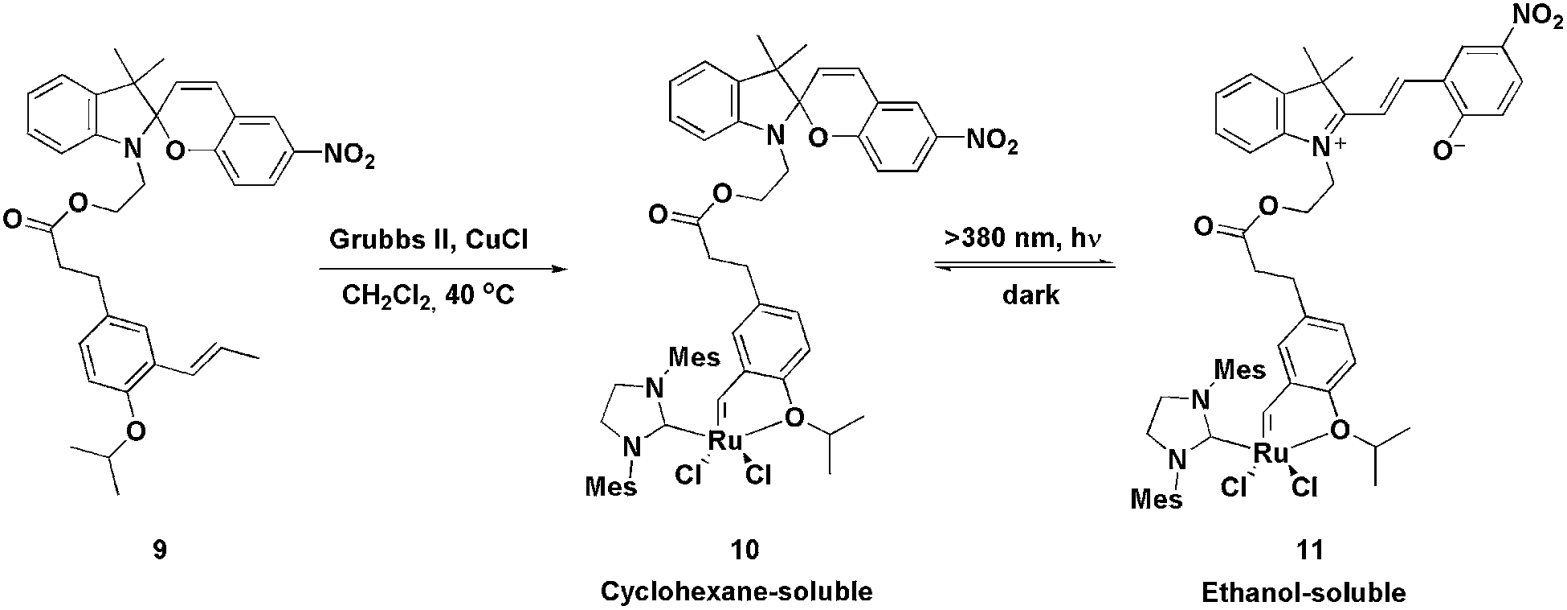
Synthesis and photoreaction of the spiro form of tagged ruthenium-photo-controlled complex **10**.

In 2010, a fashionable system for the recovery of ruthenium metathesis catalysts was described using ligand **9**.^25^ This ligand, when reacted with a second-generation Grubbs catalyst, produces the Hoveyda-type light-controlled charge-tagged complex **10**, which is a very active catalyst in the RCM of N-, S- and O-containing substituted dienes (yields ranging from 90 to 97% were obtained), even at low catalyst loadings. Moreover, this type of light-controlled tagged ligand can be switched between a non-polar and a polar phase through a tag-centred photoreaction. The photoreaction is reversible and results in drastic changes in the polarity and solubility of the ligand. In this way, after the RCM reaction, the solvent could be evaporated and the residue dissolved in a mixture of cyclohexane and alcohols (1 : 1). Thus, the catalyst and products were dissolved in the upper (cyclohexane) layer. The system was then irradiated with light (*λ* >380 nm) to transform the spiro form, **10**, into the ionic tag **11**, which completely shifted into the lower layer (alcoholic phase). The products remained in the apolar phase, which was subsequently removed. Next, CH_2_Cl_2_ was added and the mixture was kept in the dark for 3–5 min in order to re-obtain the neutral species **10** (Scheme 3). The solvent mixture was then evaporated and CH_2_Cl_2_, in conjunction with a new bath of diene, was added; six RCM cycles could be performed, retaining almost the same activity.

**Scheme 4:**
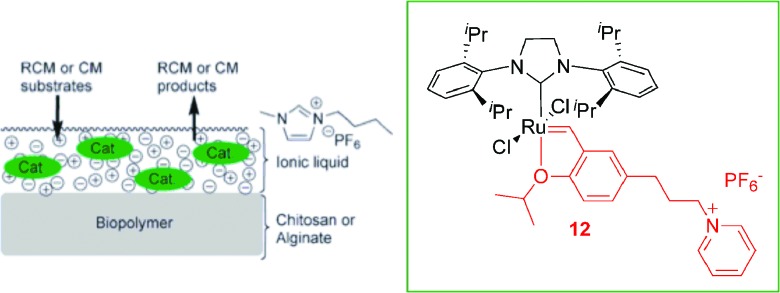
Schematic representation of the ruthenium bio-SILP. Adapted from ref. 26.

Recently, ruthenium supported ionic liquid phase (SILP) catalysts based on alginates or chitosans were synthesised and applied in olefin metathesis reactions with a high level of recyclability and reusability combined with a good reactivity.^26^ The CTL 12 and the ruthenium supported ionic liquid catalyst (Ru bio-SILP) used in this work are illustrated in Scheme 4. The reactions were performed under both mono- and biphasic conditions using the ionic liquid BMI·PF_6_. The best results were obtained in the RCM of diethyl-2,2-diallylmalonate under heterogeneous conditions with the alginate based Ru bio-SILP (at least 15 cycles could be performed with high activity ≥ 87%). The detection of ruthenium leaching by ICP-MS in the extraction phases showed low leaching of Ru throughout the cycles (200 ppm). This system was also active in the cross-metathesis reaction, with a high level of reusability (at least 12 cycles with activities up to 90%). This reusability could be attributed to the good anchoring of the catalyst in the IL phase supported on the biopolymer.

#### Metal-catalysed cross-coupling reactions

**Fig. 2:**
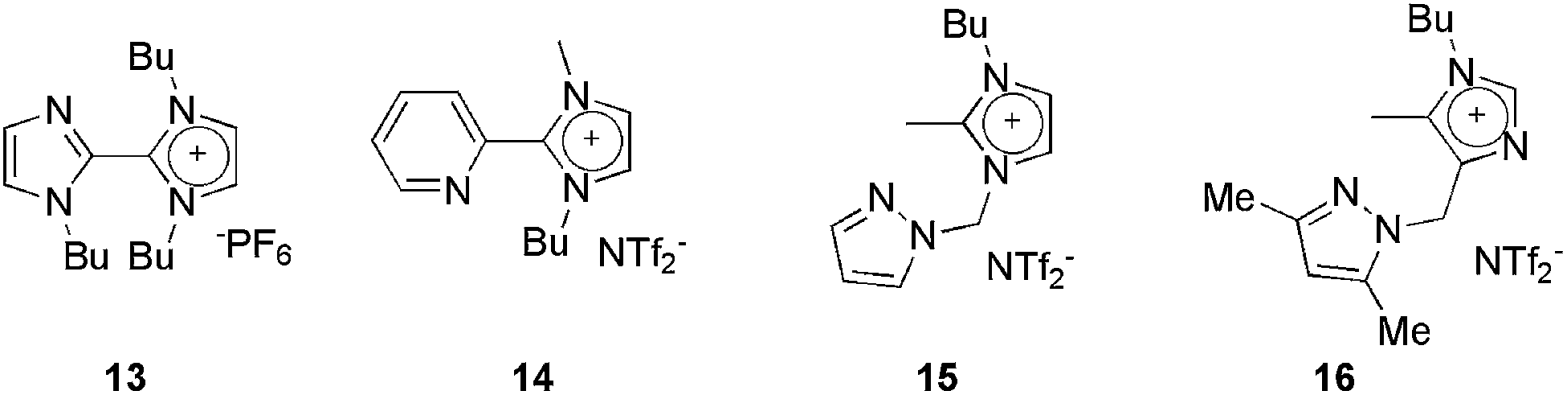
CTLs used as the solvent/ligand in palladium-catalysed cross-coupling reactions.

Several examples of using substituted imidazolium salts as both the solvent and the ligand in cross-coupling reactions have been reported (Fig. 2). In these CTLs, a coordinating imidazole, pyrazole or pyridine group was attached to either C2 or C1 of the imidazolium moiety.^27–30^ For instance, mono-charged ligand **13**, in combination with PdCl_2_, was very active in both Suzuki (yields ranging from 80 to 90%)^29^ and Heck (yields ranging from 70 to 94%) cross-couplings.^28^ In terms of reusability, for the Heck reaction, the system could be reused 9 times, even when less active aryl chloride was used. For the Suzuki cross-coupling reaction, the system could be reused 14 times without an apparent loss of activity.

The complex resulting from the reaction of **14** and PdCl_2_ was used in the Heck reaction of acrylates and styrenes with aryl iodides or bromides, using **14** as the solvent.^27^ In the reaction of PhI with *n*-butyl acrylate, a 99% yield was obtained over nine Heck reaction cycles. Moreover, yields from 49 to 94% were obtained when the same catalytic system was reused 14 times whilst varying the coupling partners.

Pyrazolyl-substituted ionophilic compounds **15** and **16** were effective when used as the solvent and ligand to stabilise the palladium species in Heck, Suzuki and Sonogashira cross-coupling reactions.^30^ In all cases, the catalysts could be reused several times without loss of activity.

**Scheme 5:**
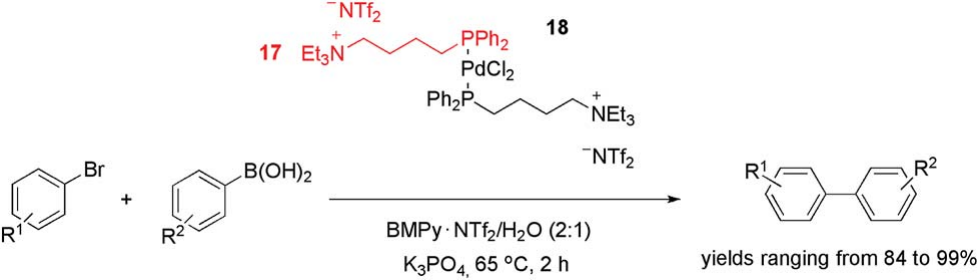
PdCl_2_-tagged phosphine used as a precursor in the Suzuki cross-coupling reaction.

In addition to nitrogen-based ligands, phosphorus CTLs have also been effectively employed in palladium-catalysed cross-coupling reactions. The tagged diphenylphosphine ligand **17**, for example, was used to produce palladium complex **18** (Scheme 5).^31^ This precursor was active in the Suzuki coupling of aryl bromides and arylboronic acids in a mixture of 1-butyl-1-methyl-pyrrolidinium bis(trifluoromethylsulfonyl)imide and water (2 : 1). With a catalyst loading of 1%, the biaryls were obtained with yields ranging from 84 to 99%. Surprisingly, when aryl iodides were used, lower yields were obtained. This result was attributed to IL anion exchange (from NTf_2_^–^ to I^–^). This hypothesis was supported by the fact that the addition of KI inhibited the conversion, whereas the addition of KBr did not cause a significant decrease in the reaction rate. In terms of reusability, six cycles could be performed without an apparent loss of activity. Moreover, no “Pd black” was observed, and <10 ppb of the metal was leached from the IL into the product during recycling.

It is important to note that, in the majority of these cases, the use of “activated” aryl iodides and bromides suggests the probable involvement of Pd(0) species, including low nuclearity Pd nanoparticles (NPs).^32^ Moreover, in the cases involving the imidazolium nucleus, it is quite probable that N-heterocyclic (NHC) carbenes are involved, at least as transient species.^33^ However, in each of these cases, the CTL is able to stabilise and retain the active catalytic species in the IL phase.

Another important aspect of Heck coupling reactions in ILs is the possibility to induce a higher degree of selectivity, because the neutral and ionic pathways of this reaction can lead to opposite regioselectivities. Indeed, the Pd complexes associated with P-containing ligands in BMI·BF_4_ promote the regioselective arylation of butyl vinyl ether, affording the α-isomer almost exclusively. Conversely, reactions performed in organic solvents afford variable mixtures of the α- and β-isomers.^34^ Unfortunately, this process has not yet been investigated using CTLs.

**Scheme 6:**
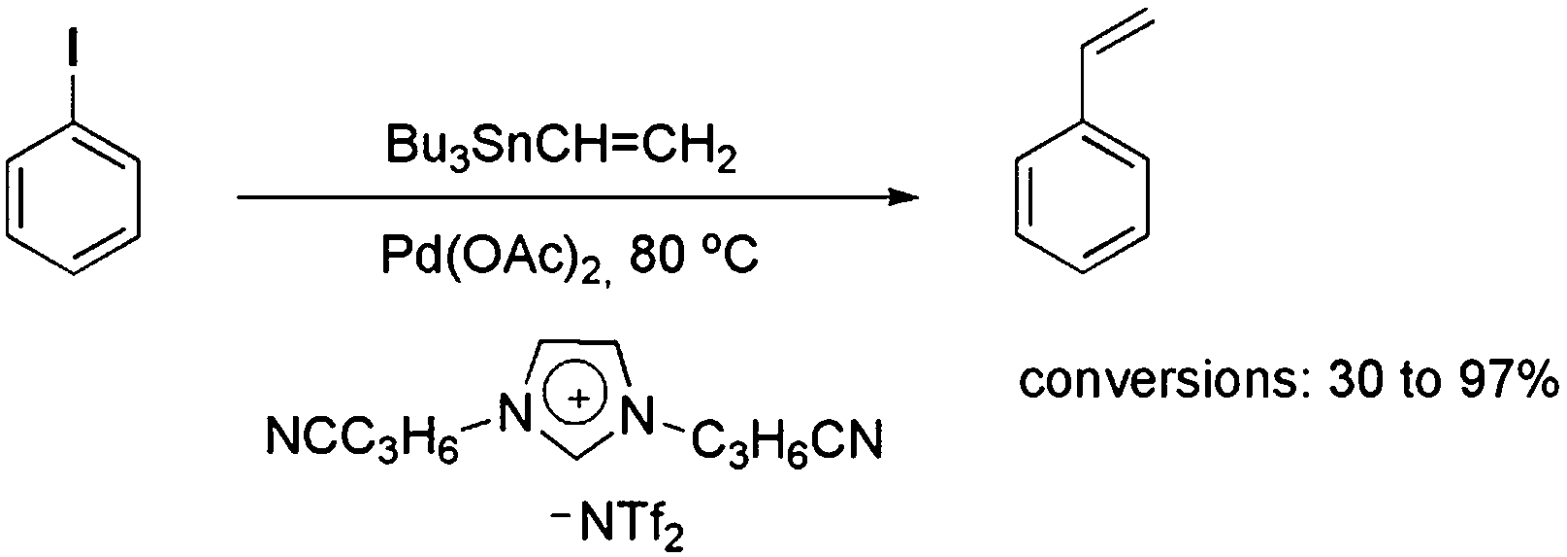
Stille reaction and the IL/ligand that was employed.

A variety of CTLs/ILs has also been used to promote better NP stabilisation, as well as to extend the catalyst’s lifetime during catalytic reactions. In this context, the catalytic activity of palladium complexes immobilised in alkyl-substituted and nitrile-functionalised pyridinium ILs was tested in the coupling of tributylphenylstannane and iodobenzene.^35^ However, the presence of [Pd(0)]_*n*_ NPs was identified after the Stille reaction. Moreover, recycling experiments showed that the functionalised IL was a superior reaction medium compared with a non-functionalised analogue, which may be associated with enhanced stabilisation of the formed NPs in the nitrile–pyridinium CTL/IL, as corroborated by transmission electron microscopy (TEM). Imidazolium-based CTL/ILs containing nitrile groups attached to the alkyl side chain were also employed in the Stille reaction (Scheme 6).^36^ In this case, a detailed study showed the influence of the relative coordination strengths of the cations and anions on the efficiency of the coupling reaction. Interestingly, NPs were only formed in some cases, and the superior conversion observed for the nitrile-functionalised IL compared with the non-functionalised one was assigned to the improved stabilisation of the NPs offered by the nitrile CTL.

**Scheme 7:**
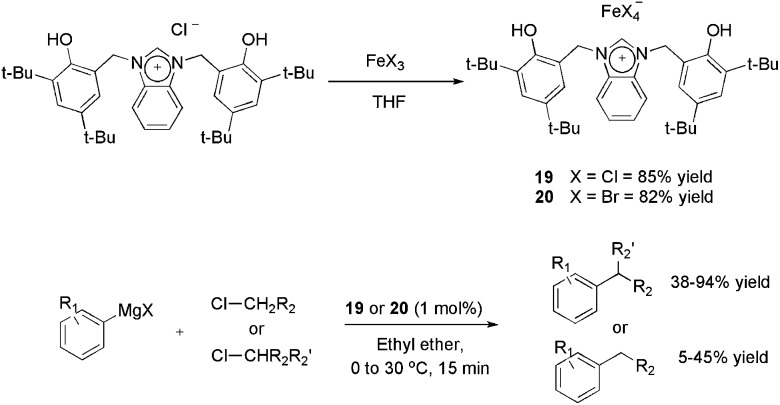
Synthesis of ionic iron–benzimidazole complexes and their application in the cross-coupling of aryl-Grignard reagents with alkyl halides.

A novel bisphenol-functionalized benzimidazolium salt was synthesised and applied in the obtainment of the air-stable ionic iron(iii) complexes **19** and **20** with 85 and 82% yields, respectively (Scheme 7).^37^ Iron-based catalysts have been employed in the alkylation of Grignard reagents^38,39^ in place of palladium and nickel complexes, because of the possibility of undesired hydride β-elimination in these complexes. However, in most reports, the more active alkyl bromides and iodides are employed as the electrophilic partner using catalyst loadings around 5 mol%. Both complexes **19** and **20** afforded cross-coupling products with reasonable to excellent yields using 1 mol% of iron and secondary alkyl chlorides as the electrophile.

On the other hand, the results using primary chlorides were unsatisfactory, considering that yields ranging from 5 to 45% were observed. In recycling experiments, **19** provided the coupling product with yields greater than 95% for six runs, however, in these experiments 5 mol% of iron (instead of 1 mol%) and a more active alkyl bromide were applied.^37^ The authors also highlighted that **19** outperforms the catalytic activity of an imidazolium analogue,^40^ especially in avoiding the formation of the β-elimination product.

#### Metal-catalysed enantioselective reactions

**Scheme 8:**
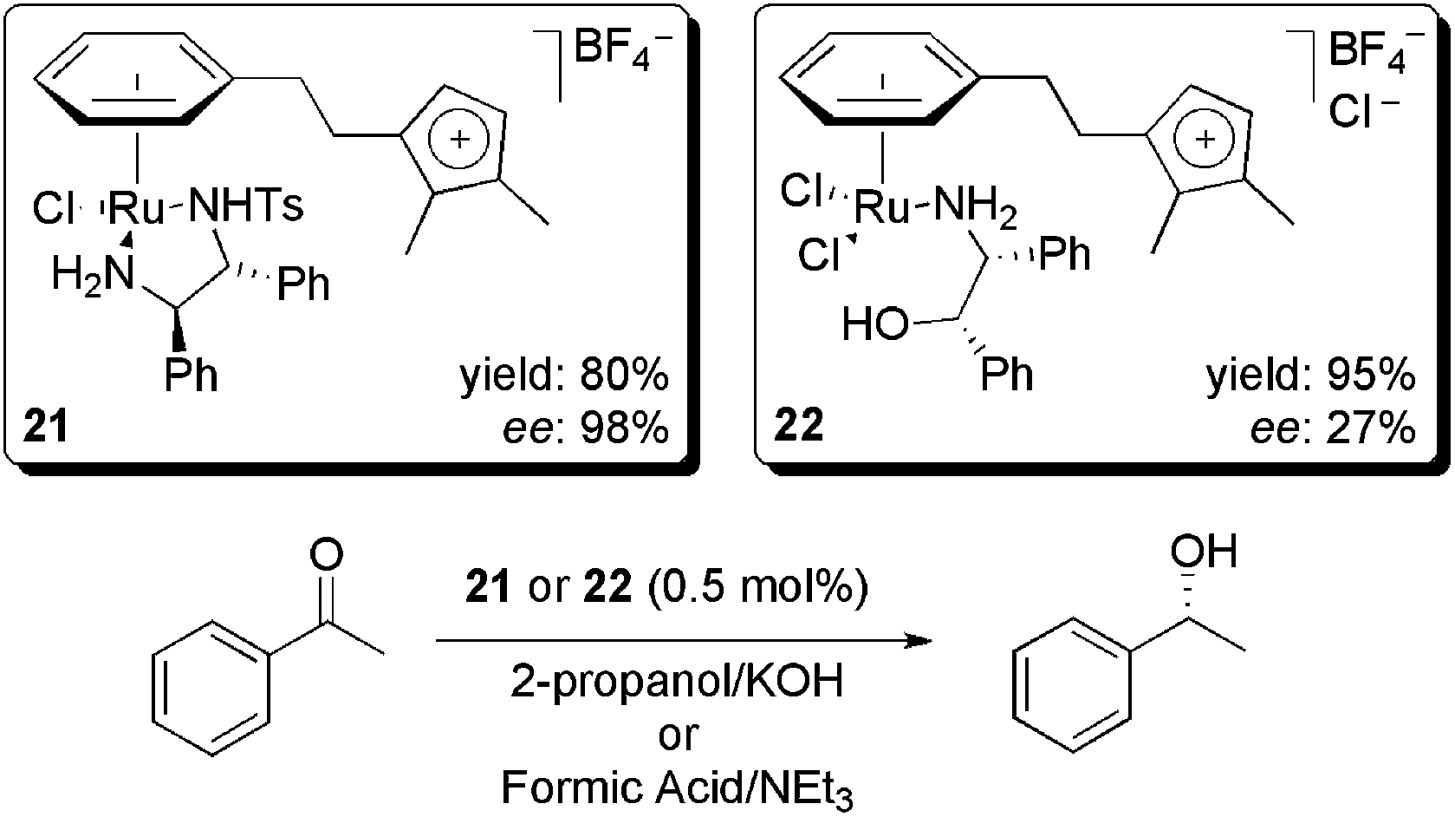
Ionophilic ruthenium complex-catalysed transfer hydrogenation reactions.

Several examples of enantioselective modifications have been reported that were catalysed by complexes with ionically tagged ligands. For example, an imidazolium cation bound to an *η*6-arene ruthenium chiral complex was evaluated and compared to neutral *η*6-*p*-cymene complexes in hydrogenation transfer reactions.^41^ Acetophenone was used as the substrate and 2-propanol or formic acid was used as the proton source (Scheme 8). For **21**, the enantiomeric excess (ee) was similar to the neutral counterpart (98%). In addition, for both **21** and **22**, the leaching was lower than for the neutral complexes when the reaction was performed in 1-butyl-2,3-dimethylimidazolium hexafluorophosphate. However, the reaction yield decreased upon recycling. This result was attributed to base-induced catalyst degradation.

**Scheme 9:**
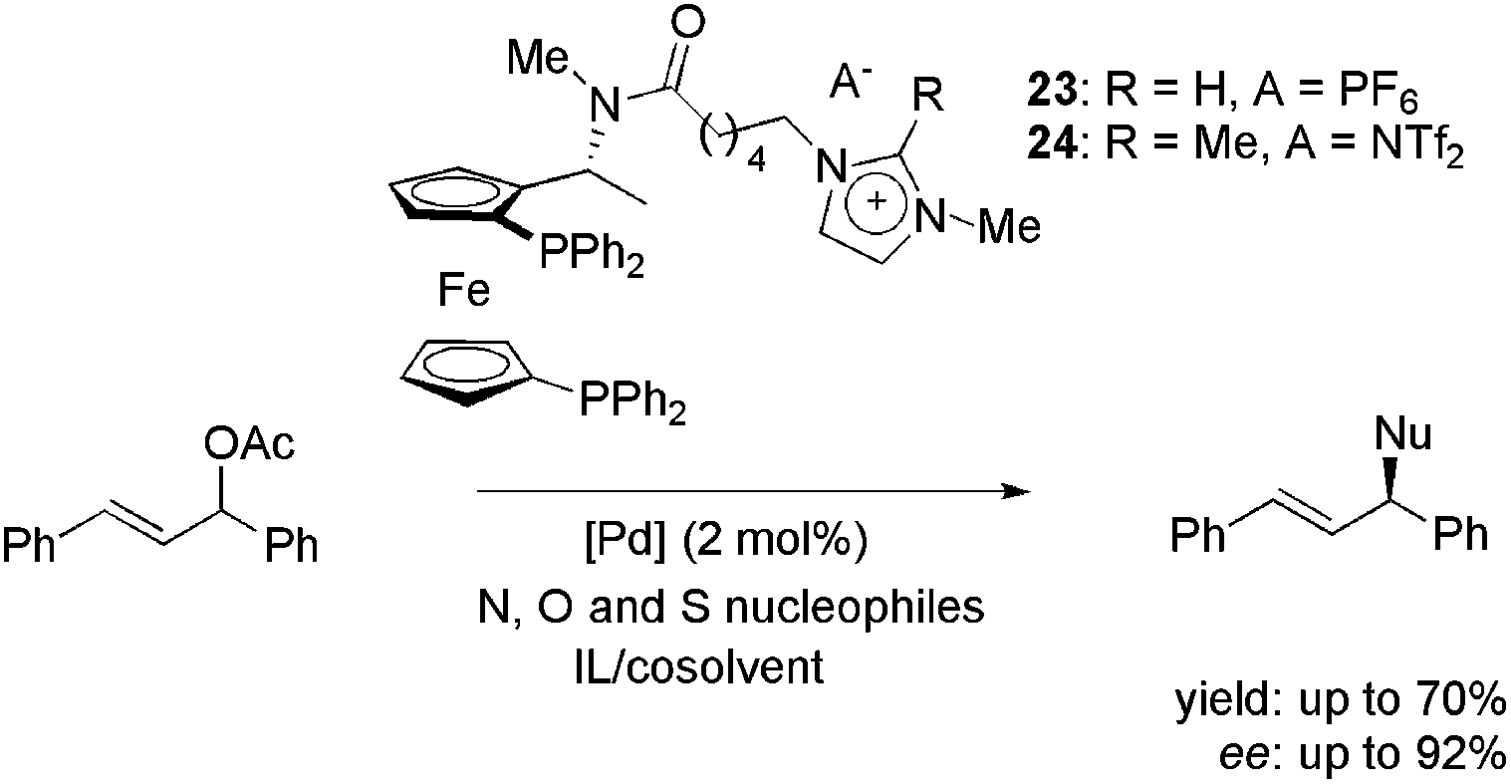
Imidazolium-tagged ferrocenyl diphosphines employed in the palladium-catalysed allylic substitution with heteroatom nucleophiles.

Imidazolium-tagged ferrocenyl diphosphines **23** and **24**, analogues of Josiphos ligands, were employed in palladium-catalysed enantioselective allylic substitution reactions with heteroatom nucleophiles (Scheme 9);^42^ potassium phthalimide, 4-methylphenolate and *p*-tolylsulfinite were also employed. For the N-containing nucleophile, the most favourable compromise between yield and enantioselectivity was obtained in a medium composed of EBI·EtOSO_3_ and CH_3_CN (70% yield and 92% ee).

For O- and S-containing nucleophiles, only modest results were obtained (41% yield with 62% ee and 81% yield with 10% ee, respectively). Moreover, when recycling experiments were performed, disappointing results were obtained. This was attributed to the high reactivity of the C–H bond at the C2 position in the imidazolium moiety of **23** under basic conditions. However, when CTL **24**, with a methylated C2, was employed, no improvements were achieved in yield or enantioselectivity.

**Scheme 10:**
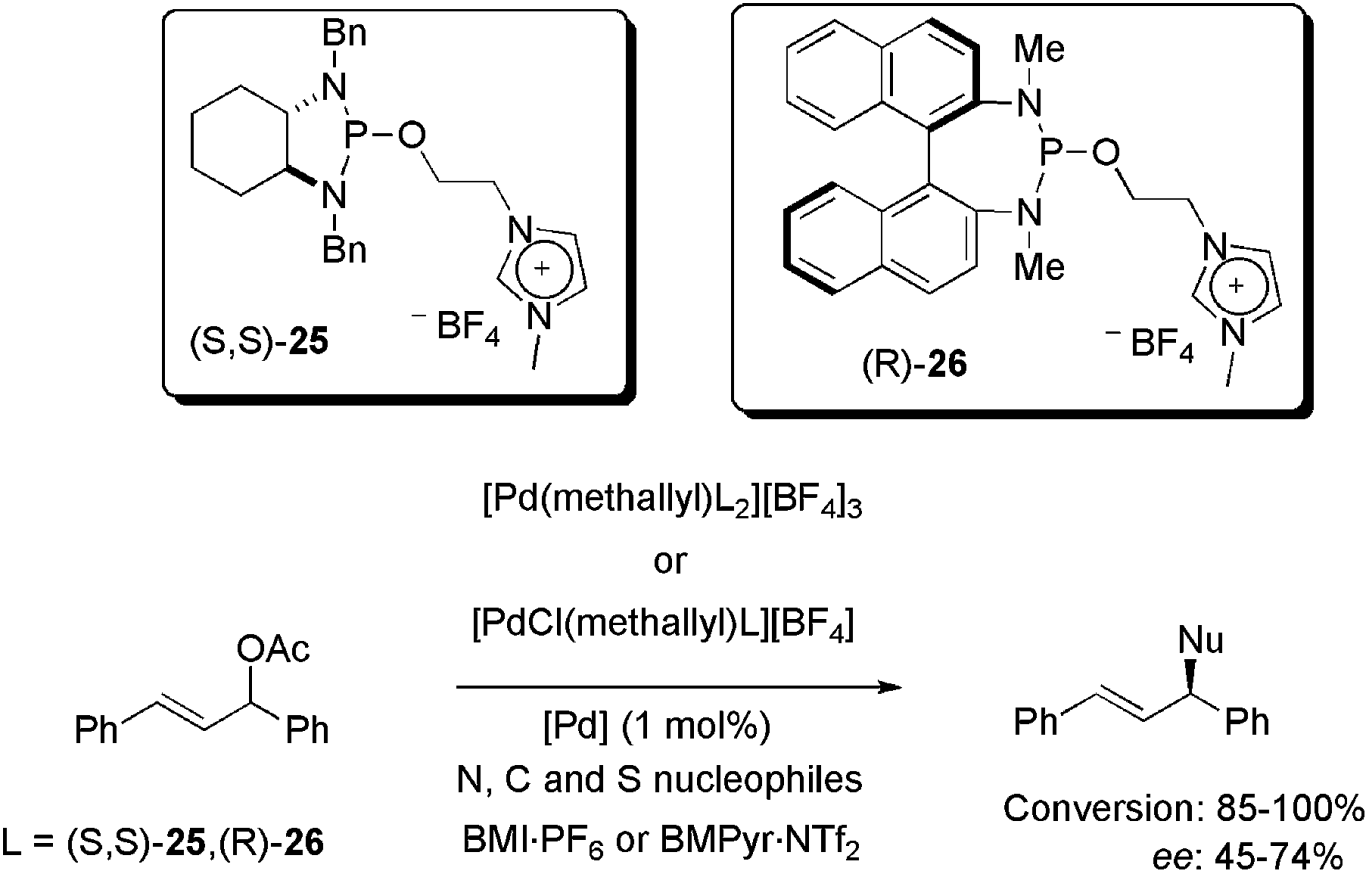
Application of allyl-palladium complexes containing an imidazolium-tagged chiral diaminophosphite ligand in N, C, and S asymmetric allylic substitution.

Recently, allyl-palladium complexes containing imidazolium-tagged chiral diaminophosphite ligands (S,S)-**25** and (R)-**26** were synthesised and also applied as catalysts in asymmetric allylic substitution reactions with heteroatom and carbon nucleophiles (Scheme 10).^43^ Complexes containing one and two units of (S,S)-**25**/(R)-**26** were tested in BMI·PF_6_ and BMPyr·NTf_2_ as reaction media. Only 1 mol% of palladium was enough to promote the substitution with up to 100% conversion for N and C nucleophiles and 85% conversion for S nucleophiles, in three hours at 35 °C. Concerning the enantioselectivity, 74% ee was obtained using benzylamine, 72% ee was observed for sodium *p*-toluenesulfinate and only 45% ee was obtained using sodium diethylmalonate. The best results, considering both reactivity and enantioselectivity, were attained using [PdCl(methallyl)**26**][BF_4_] in BMPyr·NTf_2._ In recyclability tests, unimpressive results were observed, since the conversion decreased from 75 to 55% in four runs, while the ee decreased from 75 to 58% ee over 10 reaction cycles. These results were attributed to catalyst deactivation, given that palladium was not detected in the ICP-MS analyses of the organic extractive phase. It is important to mention, however, that to the best of our knowledge, only one efficient IL-based recycling system has been reported for Pd-catalysed asymmetric allylic substitution.^44^

**Scheme 11:**
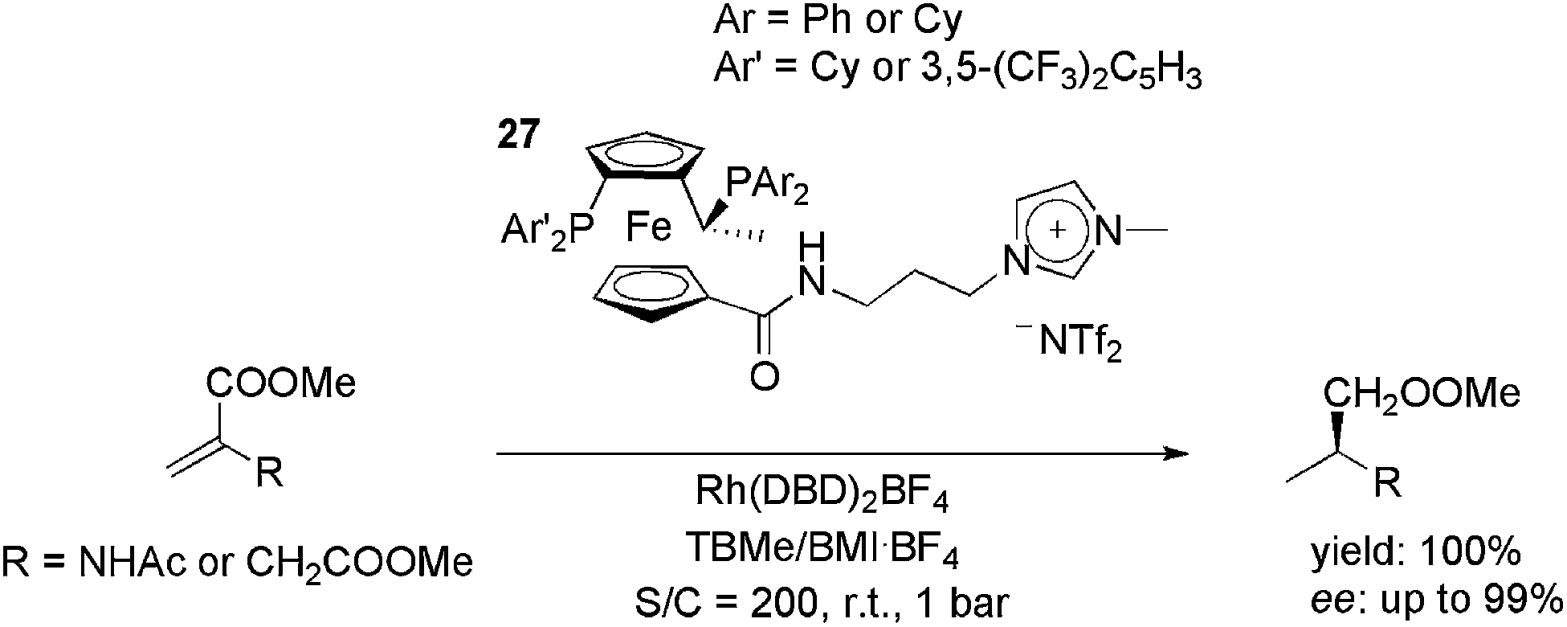
Cationic modified Josiphos ligands that were employed in rhodium-catalysed, biphasic, enantioselective hydrogenation.

Ionically tagged Josiphos analogues, **27**, were applied in the rhodium-catalysed hydrogenation of the benchmark substrates methyl acetamidoacrylate (MAA) and dimethyl itaconate (DMI) (Scheme 11).^45^ The cationic precursor, Rh(NBD)_2_BF_4_ (NBD = 2,5-norbornadiene), was used with a H_2_ pressure of 1 bar at room temperature. Initially, it was observed that the use of the appropriate reaction media was crucial for both activity and enantioselectivity. By using a biphasic system composed of *tert*-butyl methyl ether and BMI·BF_4_, total conversion and a 99% ee were obtained. In the recycling experiments, the ionic ligands kept the TOF above 1000 h^–1^ for at least five cycles, whereas with neutral Josiphos ligands the reaction rate decreased markedly upon each reuse.

**Scheme 12:**
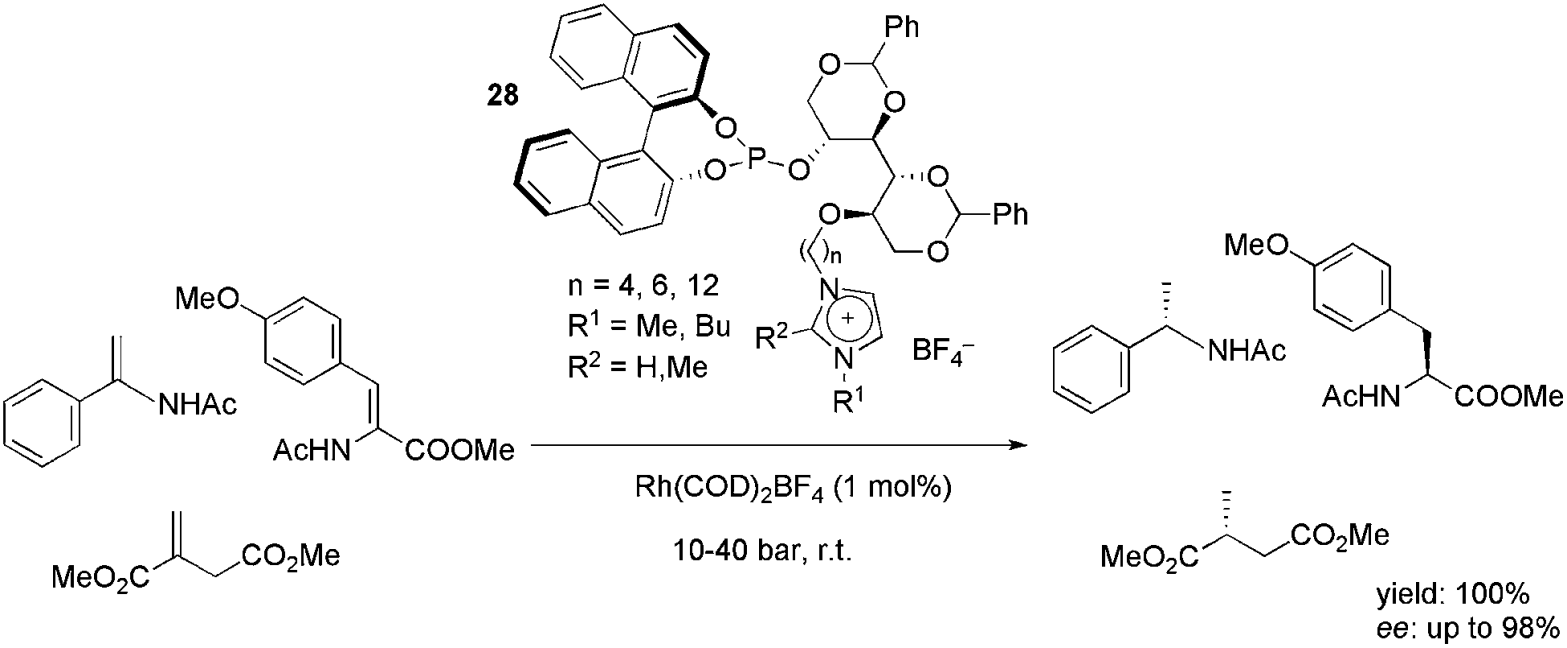
Mannitol-based charge-tagged monophosphites that were used as ligands in the rhodium-catalysed hydrogenation of olefins.

A series of ionophilic monophosphites, **28**, based on d-mannitol was synthesised and successfully applied in the rhodium-catalysed, enantioselective hydrogenation of enamides, dehydroamino acids and dimethyl itaconate (Scheme 12).^46^ The ligands provided high yields and excellent enantioselectivity in either the CH_2_Cl_2_ or biphasic IL/toluene media. The best results were obtained with the ligand bearing a six-carbon alkyl chain between the mannitol and imidazolium moieties. The recyclability of the system was tested in the BMI·BF_4_, BMI·PF_6_ and BMMI·BF_4_ (BMMI = 1-butyl-2,3-dimethylimidazolium) ILs. In all cases, up to eight recycles could be performed, keeping the yield and enantioselectivity almost constant.

**Scheme 13:**
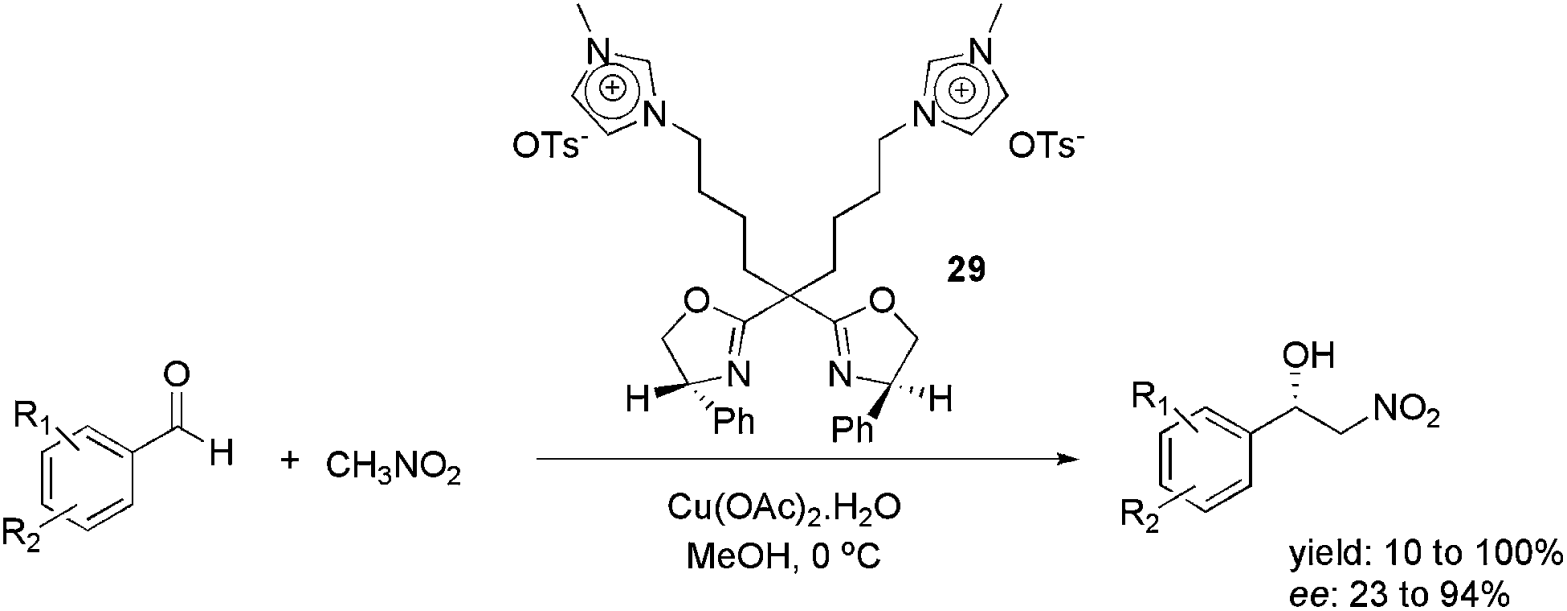
Imidazolium-tagged bis(oxazoline) ligand that was used in the copper-catalysed, asymmetric Henry reaction.

In 2012, the use of the imidazolium-tagged bis(oxazoline) ligand **29** was reported in the copper-catalysed, asymmetric Henry reaction (Scheme 13).^47^ Several substituted benzaldehydes were reacted with CH_3_NO_2_, providing the corresponding adducts. The reaction was effective in various ILs and organic solvents. However, the best enantioselectivity was obtained when the reaction was performed in MeOH. In this medium, 23 to 94% ee was obtained. Moreover, the catalyst could be reused six times, by evaporating the solvent and extracting the product with ethyl ether, without loss of activity or enantioselectivity.

#### Metal-catalysed oxidation of olefins

**Scheme 14:**
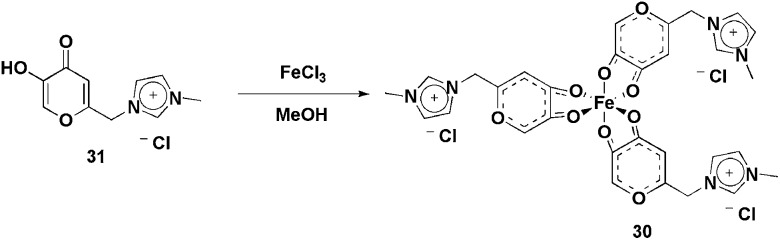
Fe(iii) complex bearing imidazolium moieties that was applied in the olefin epoxidation reactions.

An ionophilic iron(iii) complex, **30**, bearing three imidazolium moieties was synthesised (Scheme 14) by reacting **31** and FeCl_3_ in methanol.^48^ This tagged complex was used in the epoxidation of methyl oleate and vegetable oils. Yields ranging from 79 to 98% were obtained when the reactions were performed in BMI·NTf_2_, using synthetic air at 15 bar as the oxidising agent. In the oxidation of methyl oleate, the catalytic system was recycled ten times without an apparent decrease in activity. Moreover, no significant iron leaching (<2 ppm) was observed by means of inductively coupled plasma atomic emission spectroscopy (ICP-AES).

**Fig. 3:**
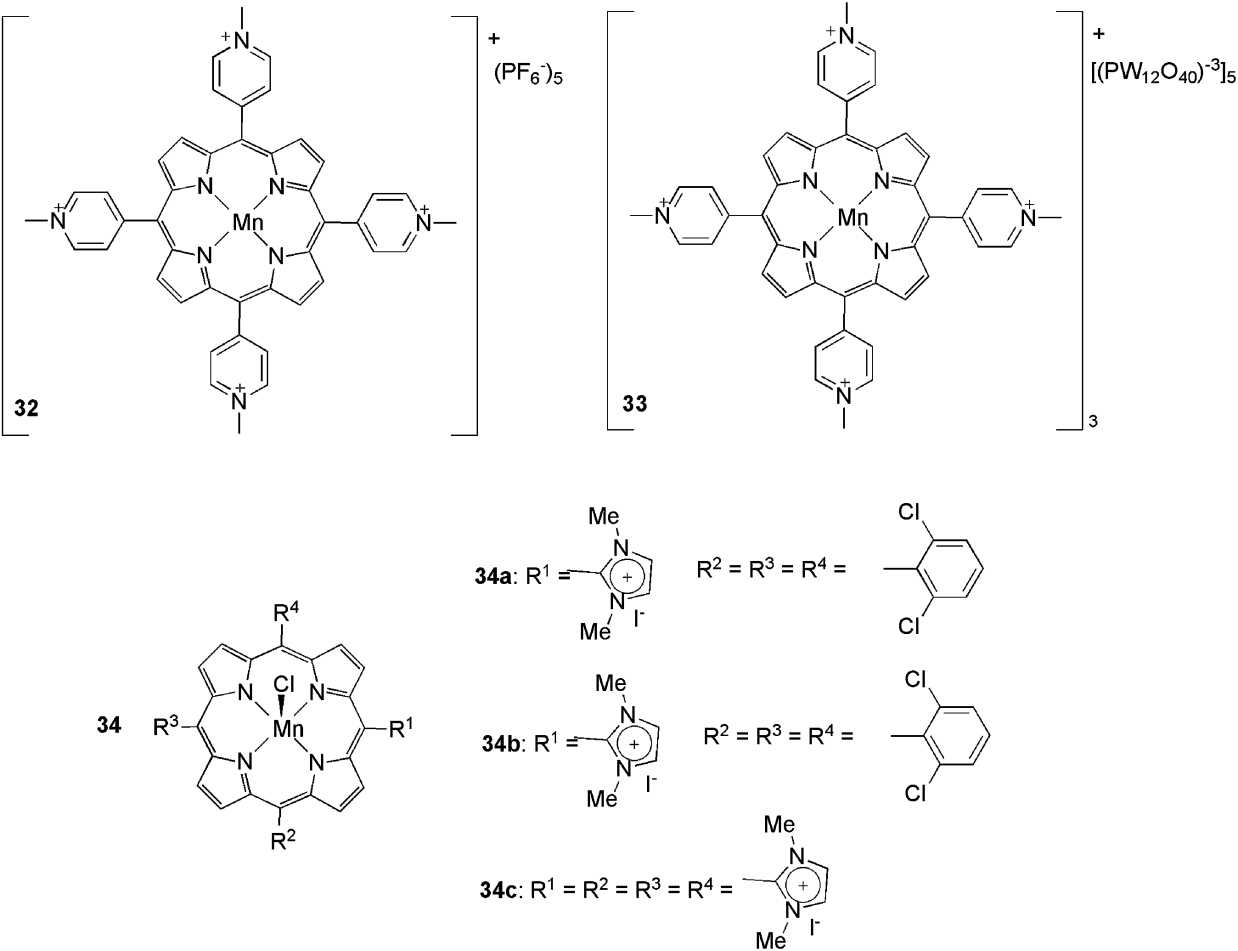
Manganese porphyrins that were used in the oxidation reactions.

**Scheme 15:**
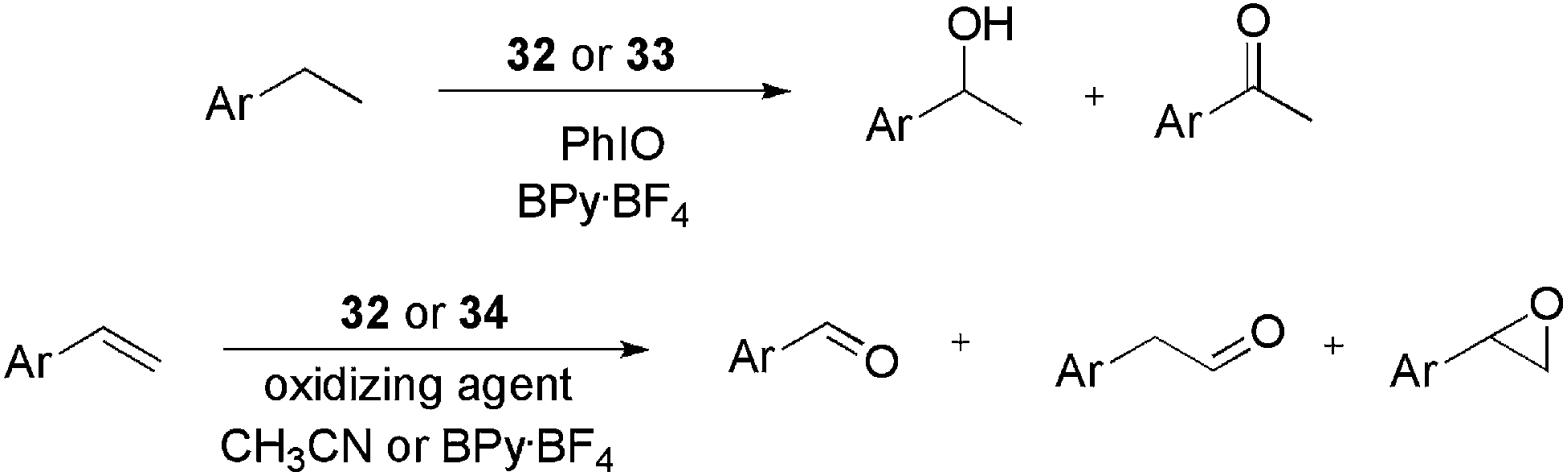
Manganese porphyrins bound to imidazolium units, which were applied to the oxidation of olefins and alkanes.

The use of ionophilic manganese porphyrins (Fig. 3) in alkane and alkene oxidation reactions has also been reported. Pyridinium-substituted catalysts **32** and **33**, for instance, were applied to ethylarene oxidation (Scheme 15).^49^ Under optimised conditions (0.4 mol% of **32**, BPy·BF_4_, and PhIO as the oxidising agent) ethylbenzene was oxidised to 1-phenylethanol and acetophenone with 71 and 29% selectivity, respectively. In recycling experiments, for both **32** and **33**, the selectivity for the ketone increased with successive reuse. In terms of activity, for **32**, the conversion decreased with reuse, whereas for **33** it remained constant over four oxidation cycles. When substituted arylethenes were used as substrates, a positive effect on the conversion was observed for the electron-donating groups. According to the authors, this result is due to the increased electron density at the α-carbon of ethylbenzene derivatives.

Catalyst **32** was also used in the oxidation of arylethenes in CH_3_CN and BPy·BF_4_.^50^ The system was active and selective for the formation of epoxides, in both media. Moreover, the recyclability of **32** was superior to that of the non-ionophilic analogue.

The synthesis of manganese porphyrins, bound to one, three or four imidazolium units (**34**), has been described^51,52^ and applied to olefin epoxidation reactions, using H_2_O_2_ as the oxidizing agent (Scheme 15). In an early investigation,^51^ ligand **34c** (olefin–catalyst = 75 : 1) was applied efficiently to the epoxidation of cyclooctene, cyclohexene, 1*H*-indene and styrene. The oxirane products were obtained with yields ranging from 95 to 100% in CH_3_CN, with acetic acid as the co-catalyst. Next, charge-tagged metalloporphyrins were compared to the neutral analogue [R^1^–R^4^ = 1,5-Cl(C_6_H_3_)] in arylethene epoxidation.^52^ All catalysts (**34a–34c**) were active in the epoxidation of styrene with a *S*/*C* ratio of 300. High conversions were attained with catalysts **34b** and **34c**. However, the best selectivity was obtained with the mono-tagged complex **34a**, which provided similar results to the neutral complex (selectivity >90%). By using substituted styrenes, the authors were able to obtain Hammett plots for the reaction. Again, the reaction was accelerated by electron-donating groups and, furthermore, the small *ρ*-values suggested no significant charge separation in the transition state.

#### Metal-catalysed olefin hydroformylation

**Scheme 16:**
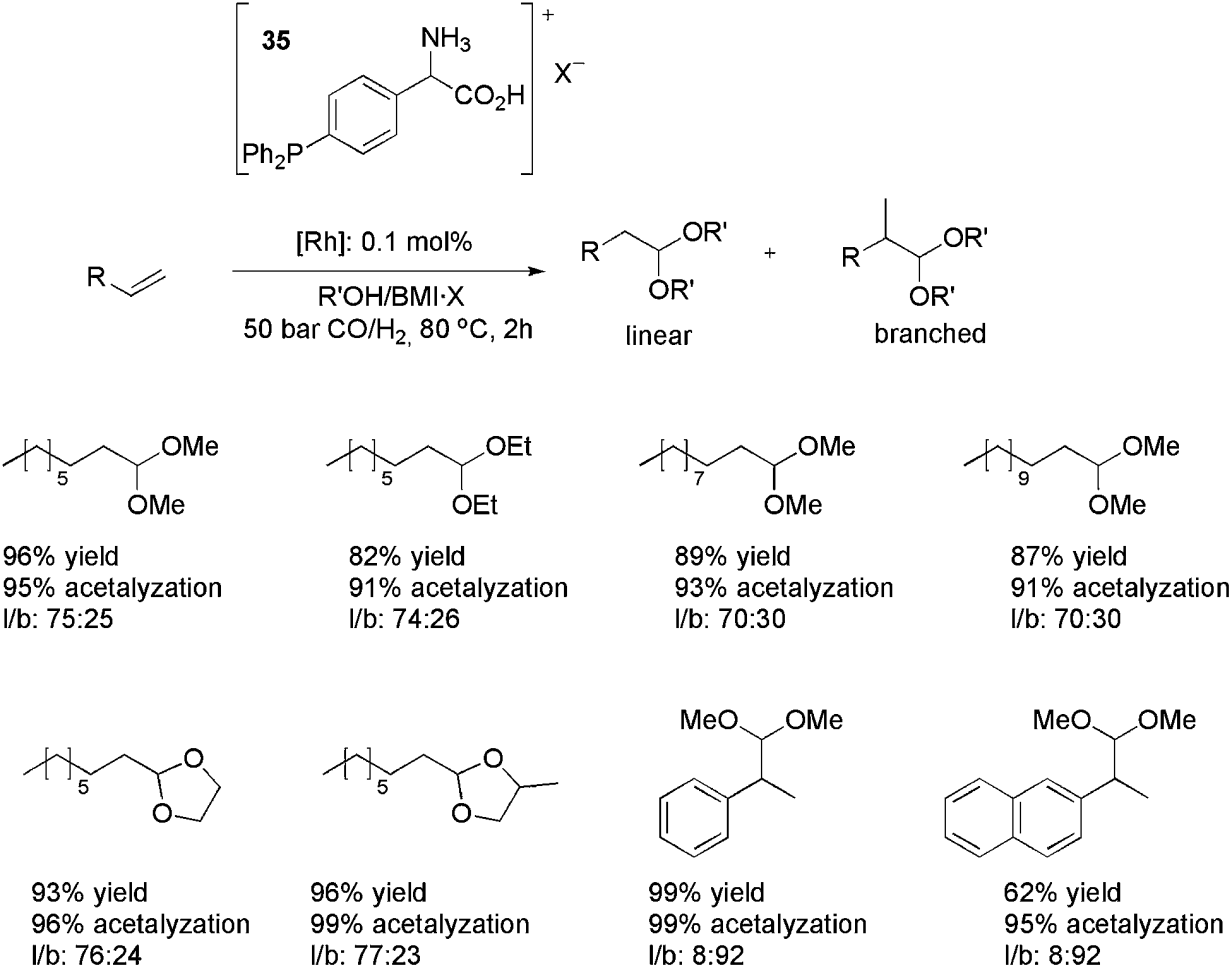
CTL **35** applied in the rhodium-catalysed tandem hydroformylation–acetalyzation of olefins in ionic liquid/alcohol media.

The cationic phosphine CTL **35** was applied in the rhodium-catalysed tandem hydroformylation–acetalyzation of olefins in ionic liquid–alcohol media (Scheme 16).^53^ Initially, 1-octene was used as the substrate model for the optimization of the ligand counteranion, catalyst loading, and ionic liquid medium. In this way, using **35**·BF_4_ and 0.1 mol% of rhodium in a medium composed of BMI·BF_4_ and methanol, the product of the hydroformylation was obtained in a 96% yield, with 95% acetalyzation and a linear–branched ratio of 75 : 25 after 2 h at 80 °C and CO–H_2_ (1 : 1) at 50 bar. Following this, other alkenes (alkyl and aryl substituted) were also hydroformylated in the presence of methanol, ethanol, ethylene glycol and propylene glycol, affording the products observed in Scheme 16. The high levels of acetalyzation were attributed to the Brønsted-acid character of the ligand **35**, since only 1% acetalyzation was observed when the CTL was substituted by PPh_3_. It is worth noting the recyclability results observed with this strategy. For a system composed of Rh(acac)(CO)_2_, **35**·BF_4_, BMI·BF_4_ and methanol, conversions greater than 98% and an average acetalyzation of 95% were observed over 12 runs. When methanol was replaced by propyleneglycol, at least 15 runs were performed without significant loss of activity. With both alcohols, the lixiviation of rhodium from the IL phase was less than 0.1%.

#### CTLs as immobilising agents in NP-catalysed hydrogenation reactions

Metal NPs have been widely used in catalysis because of their unique properties. However, it is well known that “naked” NPs are thermodynamically unstable and must be stabilised to prevent agglomeration. In this context, ionophilic ligands have been employed in catalysis to improve NP activity and stability and, most importantly, to immobilise these NPs in the ionic phase.^54^

**Scheme 17:**
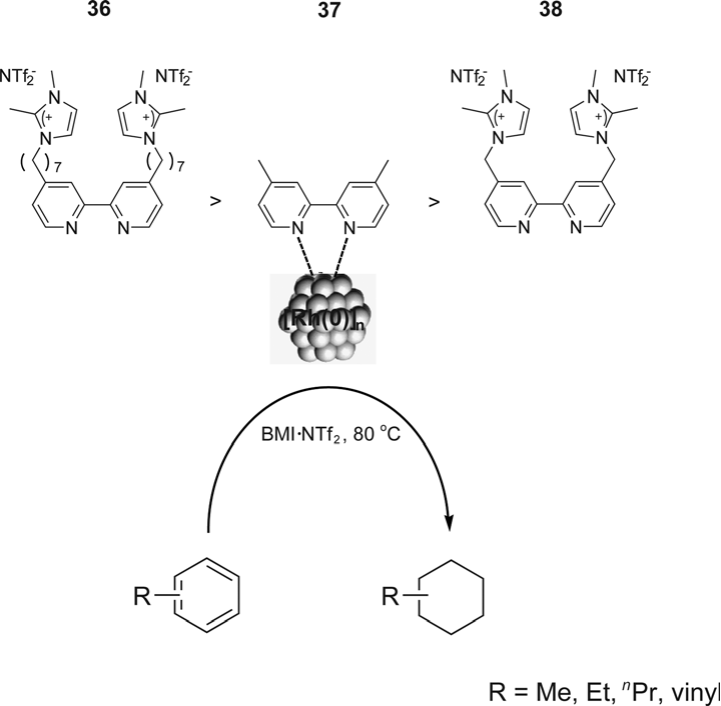
Hydrogenation of arenes, catalysed by [Rh(0)]_*n*_ NPs stabilised by CTLs in ILs, and the activity observed for the different ligands.

The catalytic activity was strongly influenced by the structure of the CTL stabiliser employed in arene hydrogenation with [Rh(0)]_*n*_ NPs, following the trend **36** > **37** > **38** (Scheme 17).^55^ The [Rh(0)]_*n*_ NPs stabilised by **36**, containing a C7 alkyl chain between the bipyridine and the imidazolium group, showed the highest activity compared to the ligand with only a methylenic group between the bipyridine and the ionophilic group **38**. This may be caused by a weaker interaction between the ligand **38** and the NP surface, which decreases the binding affinity of this ligand and promotes the decomposition of the NPs under the reaction conditions.

**Scheme 18:**
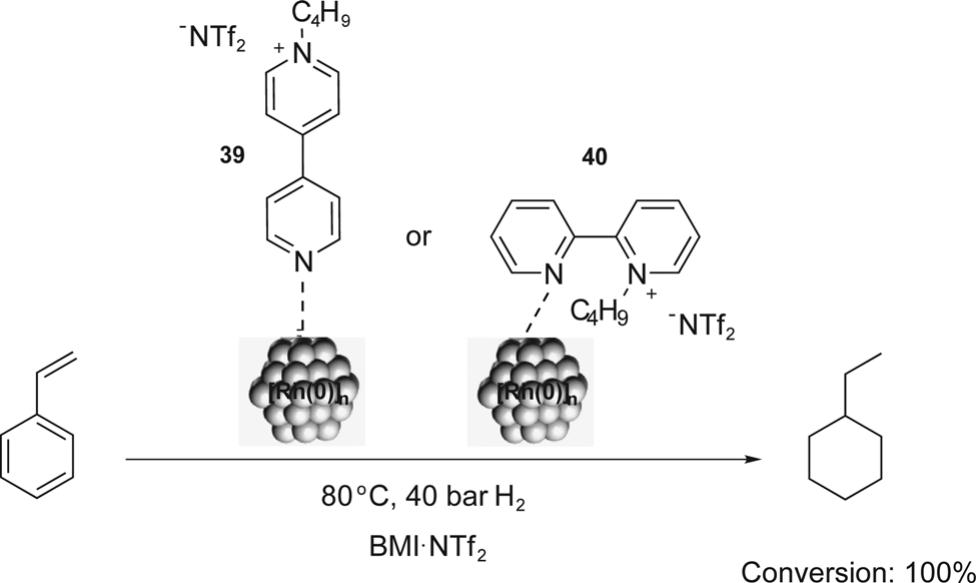
Hydrogenation of styrene catalysed by [Rh(0)]_*n*_ NPs, which are stabilised by the bipyridinium CTLs **39** and **40**.

Similarly, bipyridinium CTLs (**39** and **40**) were employed in styrene hydrogenation using [Rh(0)]_*n*_ NPs.^56^ The total conversion of styrene into ethylcyclohexane was observed and the quantitative hydrogenation of the aromatic ring was attributed to the monodentate coordination of the ligand to the NP (Scheme 18). Furthermore, NPs with small sizes (around 2.0–2.5 nm) and good stabilities were obtained in the presence of charged ligands. Comparable results were obtained in arene hydrogenation using [Rh(0)]_*n*_ NPs stabilised by imidazolium-functionalised bipyridine derivatives in BMI·PF_6_.^57^ Conversions of up to 85% were obtained for the total hydrogenation of benzene and toluene.

**Scheme 19:**
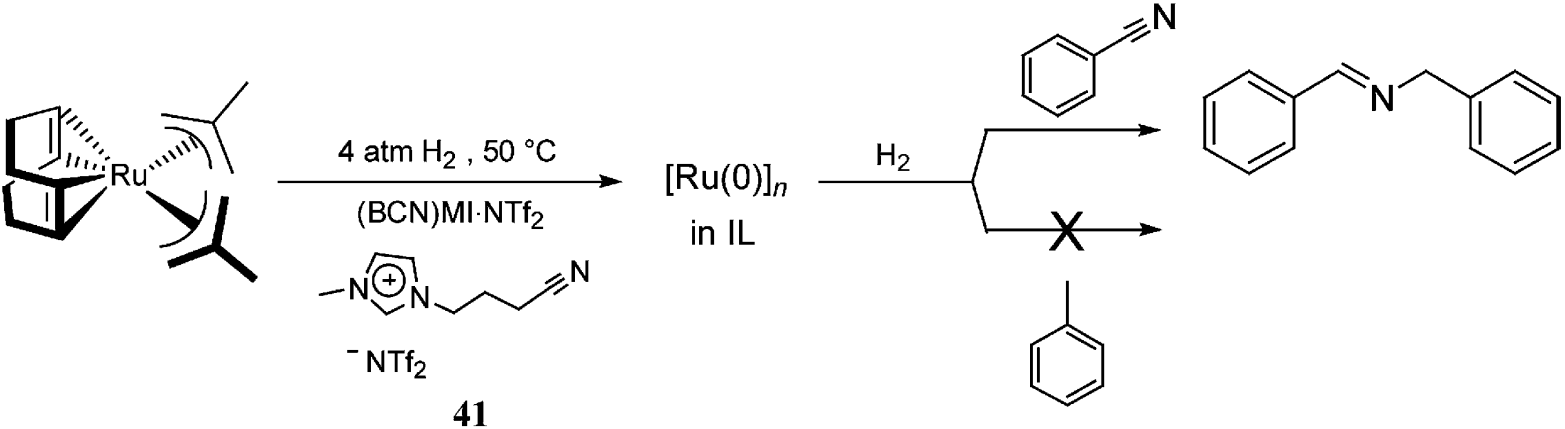
Selective hydrogenation of benzonitrile, catalysed by [Ru(0)]_*n*_ NPs in a nitrile-functionalised IL.^54^

In the same context, functionalised ILs have been employed as the CTL/solvent in catalysis to improve the stability of the NPs. [Ru(0)]_*n*_ NPs synthesised in nitrile-functionalised ILs display unusual selectivities towards the hydrogenation of nitrile-containing aromatic compounds.^58^ [Ru(0)]_*n*_ NPs dispersed in (BCN)MI·NTf_2_ [(BCN)MI = 1-butyronitrile-3-methylimidazolium], **41**, exclusively hydrogenate nitrile groups instead of arenes, which are typically hydrogenated by [Ru(0)]_*n*_ NPs in non-functionalised ILs^59^ (Scheme 19). It is plausible that, in these systems, the nitrile group of the IL is strongly coordinated to the ruthenium surface, which only allows the nitrile group of the substrate access to the NP surface, thereby avoiding arene coordination. Thus, it is possible to state that the presence of a CTL, effectively, modulates the selectivity of the catalyst.

**Scheme 20:**
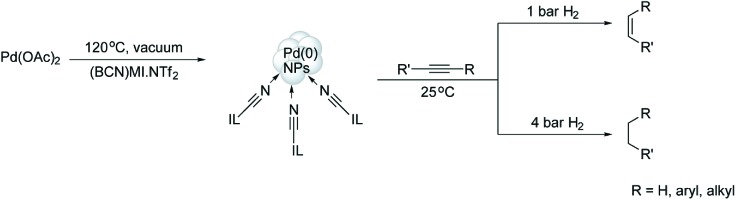
Hydrogenation of alkynes, which is controlled by a variable hydrogen gas pressure.^60^

The selective hydrogenation of alkynes to (*Z*)-alkenes, catalysed by [Pd(0)]_*n*_ NPs (7.3 nm) stabilised in the CTL/IL **41**, was also recently reported by our group.^60^ Notably, 3-hexyne was partially hydrogenated to the corresponding (*Z*)-olefin without any trace of the isomerised by-product. The lack of isomerisation indicates that the [Pd(0)]_*n*_ NPs exhibit typical behaviour and possess surface-like (multi-site) catalytic properties. Moreover, it is possible to tune the selectivity towards alkenes or alkanes, depending on the hydrogen pressure employed (Scheme 20). These results are probably related to the low hydrogen gas solubility in ILs and the associated mass-transfer/diffusion limitations. It is expected that, at low pressures, there is a minimal amount of hydrogen dissolved in the IL, affording the alkene product; on the other hand, increasing the gas pressure results in more hydrogen being present in the IL phase to yield total hydrogenation (*i.e.*, the alkane product).

### CTLs as probes for detecting reaction intermediates through ESI-MS

In the last decade, ESI-MS has become a fundamental technique for the detection of intermediates in organometallic catalysis.^21,61–63^ This method permits the transfer of ions from solution into the gas phase, thus allowing the sampling of dissolved, charged organometallics *in situ*. By using this methodology, important information concerning the catalytic cycle of a plethora of reactions could be obtained (for instance cross-coupling reactions,^64–66^ the Wacker oxidation,^67^ hydrosilylation,^68^ hydrogenation,^69^ *etc.*), including air- and moisture-sensitive ones.^70^ However, ESI represents a “gentle” ionisation method, and only charged intermediates are detectable in ESI-MS. Therefore, reactions that proceed through a neutral mechanism are not likely to be detected properly. Moreover, the common ionisation techniques, protonation/deprotonation, can affect the nature of the catalytic system under investigation. To overcome this limitation, some strategies have been employed, including (i) the formation of charged adducts^71^ and (ii) the use of charge-tagged substrates (this strategy has been applied successfully for olefin metathesis,^20^ cross-coupling reactions,^19,72,73^ the Pauson–Khand [2 + 2 + 1] cycloaddition^74^ and other catalytic processes^75^). However, both (i) and (ii) limit the scope of the substrates employed. An alternative is to use ionically marked ligands. Here, we describe interesting examples of the use of CTLs as probes to detect intermediates in catalysis. Note that although charged metal compounds behave straightforwardly in ESI-MS, low fragmentation conditions should be used *i.e.* a low cone angle voltage in order to observe parent ions or derivatives. For the details, scope and limitations of the use of ESI-MS readers are invited to consult the specialized literature.^76–79^

#### Olefin metathesis

**Scheme 21:**
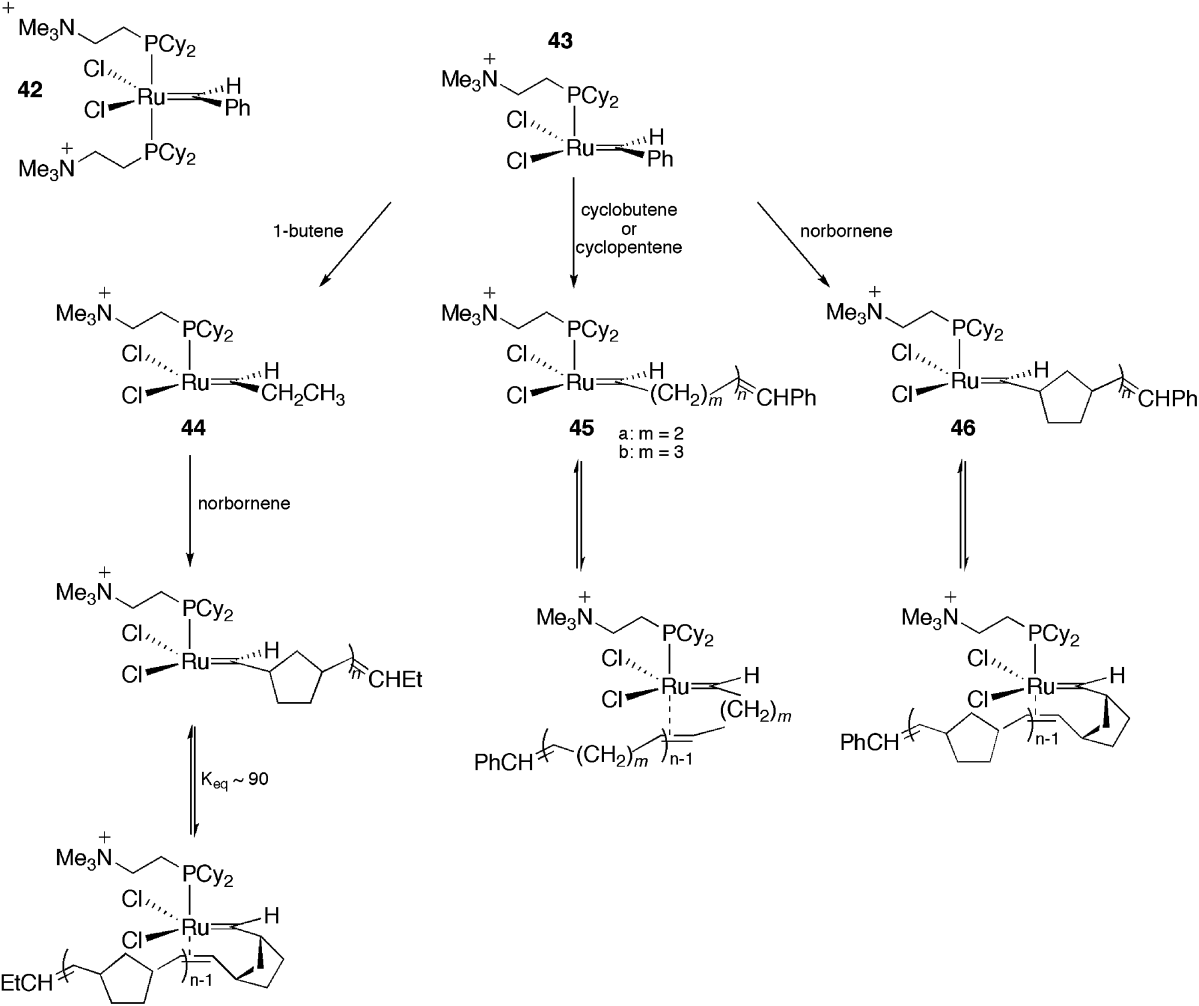
CTLs in the ESI-MS detection of olefin metathesis intermediates.

The pioneering work on CTLs to be used in the detection of intermediates through ESI-MS was published by Chen and co-workers in 1998.^80^ The authors used the charged-tagged ruthenium complex **42** as a probe in cross-metathesis (with 1-butene) and ROM (with cyclobutene, cyclopentene and norbornene) reactions (Scheme 21). Initially, the signal of **43** was observed in the gas phase, corresponding to the de-coordination of a phosphine ligand. Next, after the addition of the olefins, the metathesis adducts **44–46** were observed in the positive-ion ESI-MS [ESI(+)-MS] spectra. Adducts with additional olefin units (up to three) were also observed. The ROM of the first cycloalkene unit was much easier than the subsequent additions. This finding was attributed to the π-complexation of the penultimate double bond to the ruthenium centre.

**Scheme 22:**
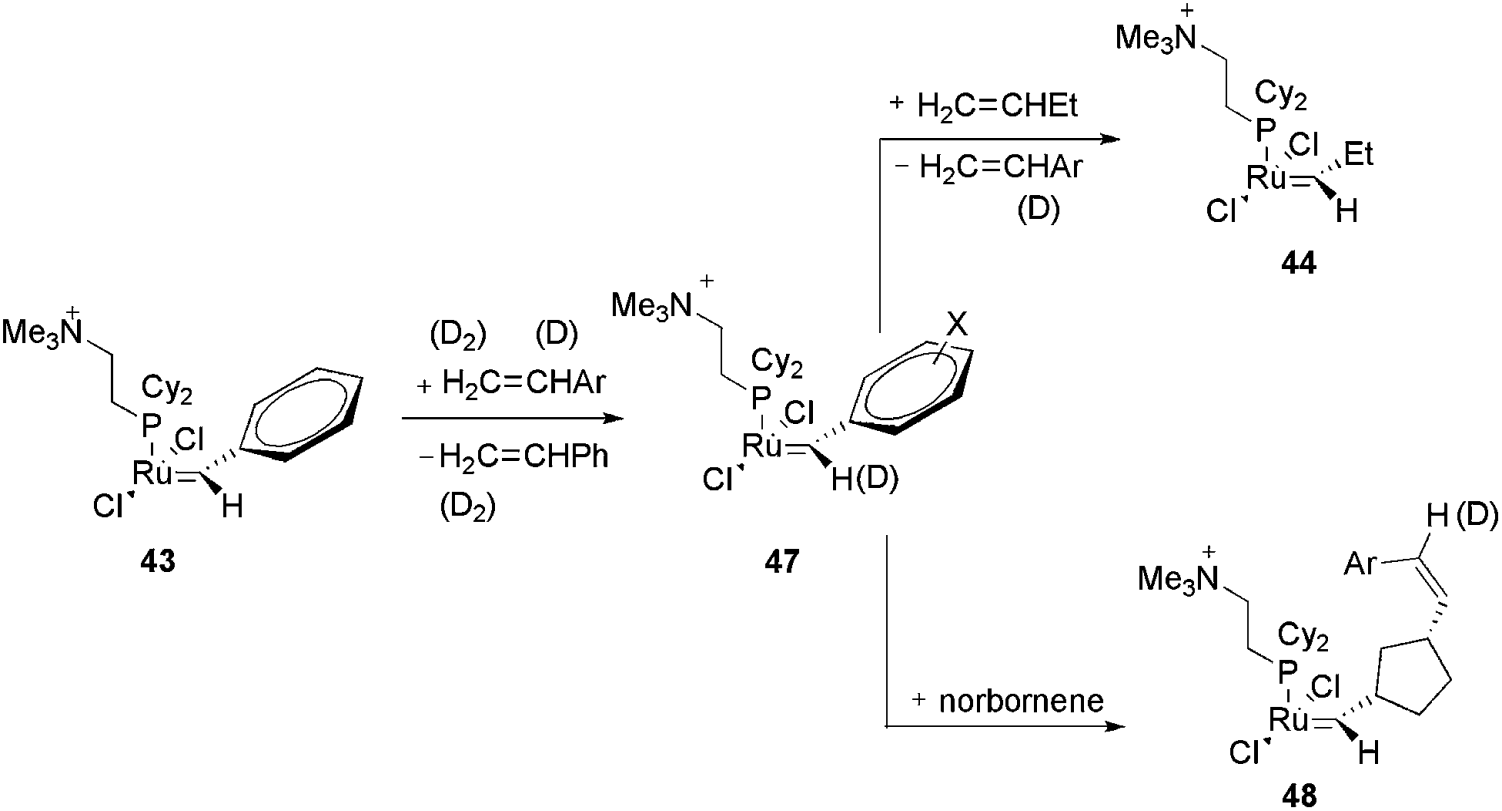
Reaction of the electron-sprayed intermediate **43** with substituted styrenes, generating **47** and subsequently **44** and **48**.

In another work with the CTL **43**, the same authors evaluated the effect of the carbene substituent and the isotope effect on the metathesis rate.^81^ Initially, the electron-sprayed intermediate, **43**, was reacted with substituted styrenes, generating **47** (Scheme 22). The reaction rate of **47** with propene and norbornene was measured. A modest acceleration of the acyclic metathesis reaction was observed in the presence of the electron-withdrawing groups on the aryl group of the substituted ruthenium benzylidene. Moreover, the carbene complex formed from **47** and styrene-d_8_ exhibited secondary deuterium kinetic isotope effects for the acyclic metathesis of 1-butene and the ROM of norbornene in the formation of **44** and **48**, respectively.

**Scheme 23:**
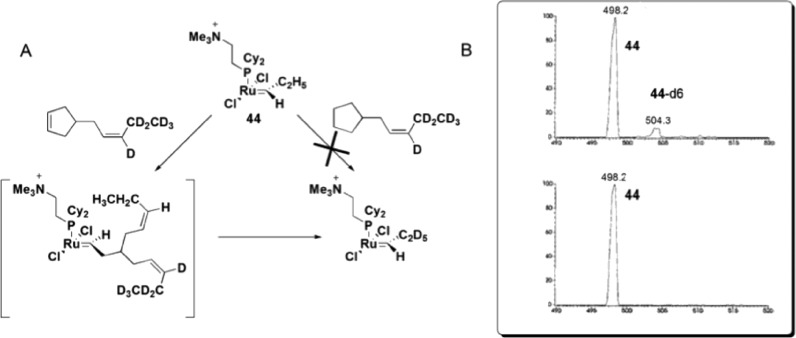
(A) Reaction of **44** with 4-[(*Z*)-2-pentenyl-3,4,4,5,5,5-d6]-cyclopentene or 4-[(*Z*)-2-pentenyl-3,4,4,5,5,5-d6]-cyclopentane. (B) Top: ESI(+)-MS spectra of the reaction of **44** with a cyclopentene derivative, with the detection of **44**-d6. Bottom: ESI(+)-MS spectra of the reaction of **44** with a cyclopentane derivative, with no detection of **44**-d6. Adapted from ref. 81. Copyright (2000) American Chemical Society.

Remarkable findings, concerning the reversibility of ROM, were obtained with ESI(+)-MS experiments for bifunctional substrates, which offer the product of a ROM reaction the chance to undergo a non-degenerate RCM to form a complex that is isotopically distinguishable from the original complex in MS. The cyclopentane derivative does not react with **44** under the relatively mild conditions in this experiment; the cyclopentene derivative, instead, leads to approximately 10% conversion of **44** to **44**-d6 (Scheme 23), indicating that the ROM of cyclopentene had occurred.

The same charge-tagged intermediate, **42**, was also used to study RCM through ESI(+)-MS experiments.^82^ Again, **43** was generated and, after a gas-phase reaction with ethylene, a charged, 14-electron, methylene ruthenium species was detected. Concerning the reactivity of N(Boc)(allyl)_2_ and other diallyl compounds in RCM, it could be confirmed that all the monomolecular steps in the catalytic cycle of the RCM, following olefin coordination, are fast, and no intermediate can be accumulated to become detectable by ESI-MS. Additionally, alkylidene trialkylphosphane and phosphonium salts were easily detected and attributed to degradation products.

#### Cross-coupling reactions

The actual mechanism of the copper-catalysed coupling between phenols/amines and aryl/vinyl halides (Ullmann coupling) is still under discussion. It remains unclear whether carbon–halide bond activation occurs through an oxidative addition, Cu(i)–Cu(iii), process (as demonstrated in theoretical studies,^83,84^ reactions with well-defined Cu(iii) compounds^85–87^ and radical-detection tests^88,89^) or *via* a radical SET/IAT Cu(i)–Cu(ii) pathway (as shown in theoretical studies^90^ and ESI-MS experiments combined with radical-scavenger tests^91^). Another point of discussion is the putative formation of a nucleophilic copper species, preceding the organic-halide activation.

**Scheme 24:**
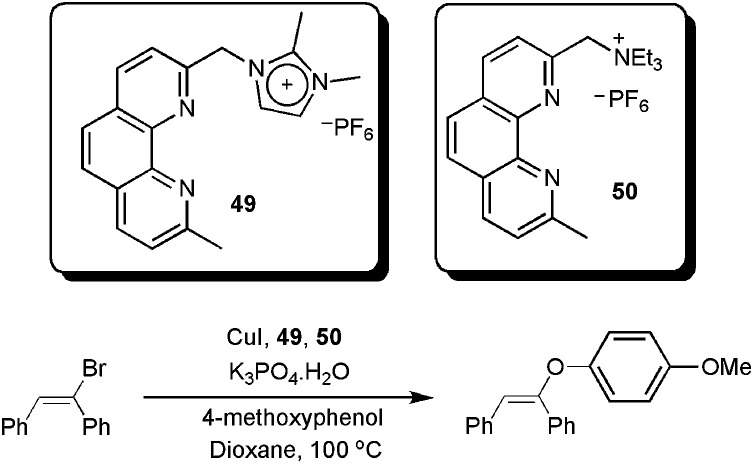
CTLs **49** and **50** as probes to detect intermediates in the copper-catalysed Ullmann vinylation.

We recently described the synthesis and use of the phenanthroline-based ligands **49** and **50** in ESI(+)-MS experiments.^92^ These cationic ligands were used as probes to detect the intermediates in the copper-catalysed coupling of (*E*)-bromostilbene with phenols (Scheme 24).

**Fig. 4:**
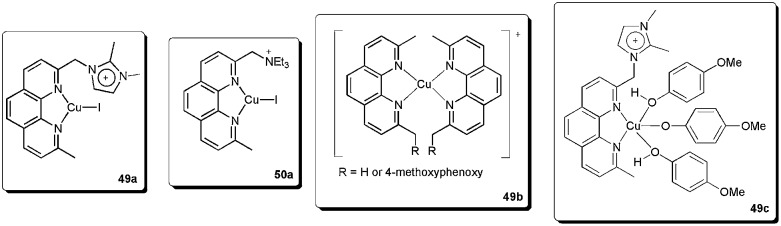
Intermediates detected in ESI(+)-MS.

In this way, we were able to detect various intermediates (Fig. 4). Initially, intermediates **49a** and **50a**, corresponding to **49** and **50** coordinated to copper iodide, were observed. After the addition of phenol, base and vinyl bromide, various resting-state intermediates could be detected. Intermediates **49b**, in which a benzylic cleavage of the ligands had occurred, were similar to those described as very active catalysts in the arylation of phenols, even at very low concentrations of copper (10 ppm).^93^ Another important intermediate was **49c**. In this species, the ionophilic probe was not cleaved and it contained a Cu–OAr bond. The formation of Cu-nucleophile species has been described in the arylation of phenols^94^ and for other nucleophiles.^84,89,95–98^ These species have been reported to be capable of reacting with aryl halides under mild conditions.^84,89,94–98^ Moreover, **49c** also indicates that the phenol moiety can act as a nucleophile as well as a ligand.

Based on the results obtained from ESI(+)-MS, preliminary kinetic studies and the radical-scavenger experiment, we could propose viable mechanistic pathways for the copper/1,10-phenanthroline-catalysed vinylation of phenols. At first, the coordination of the ligand to CuI takes place, providing a (phen)CuI species, which was detected as **49a** and **50a** in the ESI(+)-MS. Thereafter, an intermediate [(phen)_2_Cu]^+^ is formed. The formation of this intermediate was supported by the detection of **49b**. Next, the Cu–OAr intermediate was detected as **49c**, which is reported to be able to react with the organic halides.^94^ In terms of vinyl halide activation, as the radical pathway is not operational, we could argue in favour of an oxidative addition to form a Cu(iii) intermediate. Although these species are not common, arylcopper(iii) complexes have recently been identified,^85,99,100^ and arylcopper(iii) intermediates containing nitrogen ligands are calculated to be kinetically accessible under the reaction conditions.^89^

**Scheme 25:**
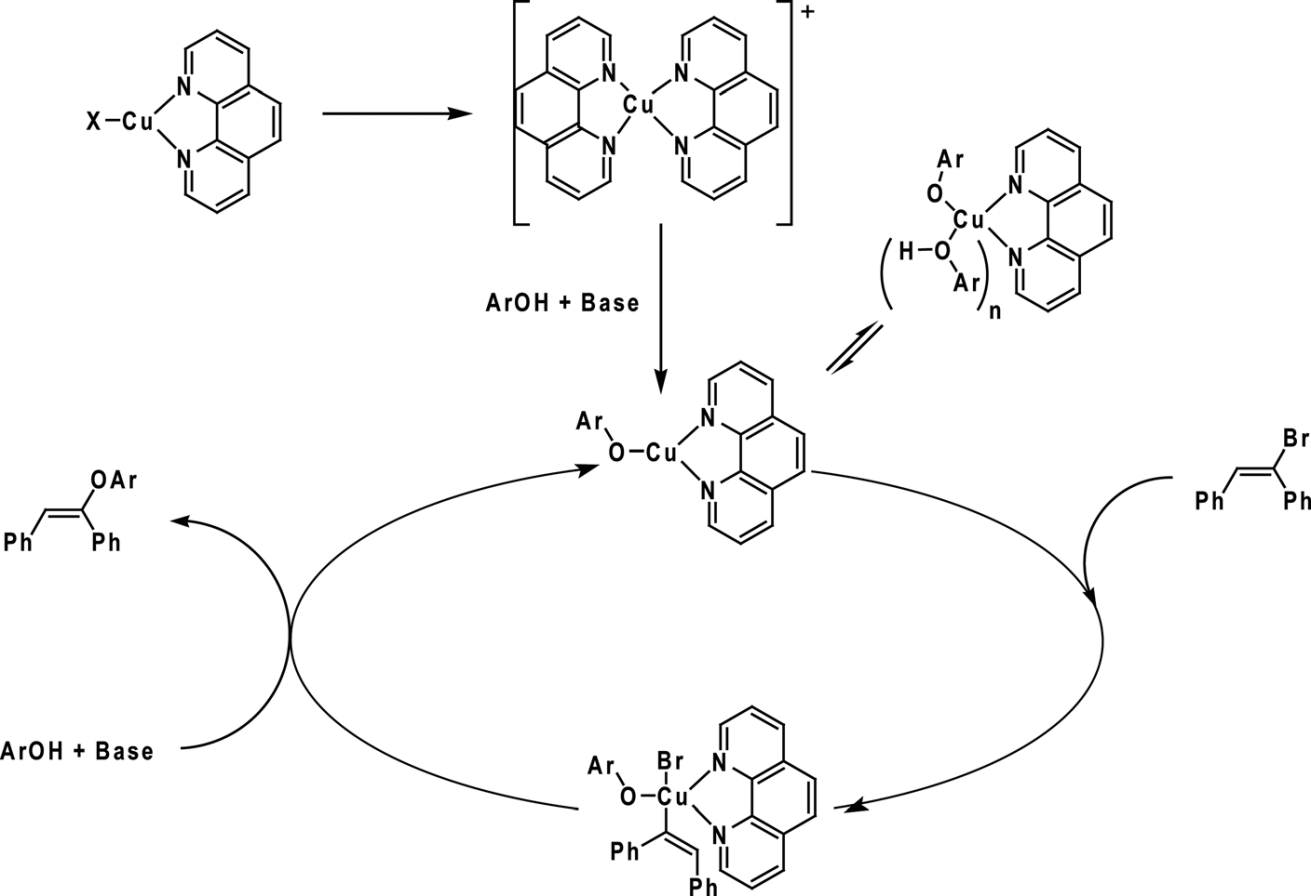
Catalytic cycle proposed for the copper-catalysed vinylation of phenols. Adapted from ref. 92.

Then, the [(phen)-CuOAr] intermediate would be reformed by a putative reductive elimination, followed by the attack of the phenoxide to form the copper species (Scheme 25).

**Scheme 26:**
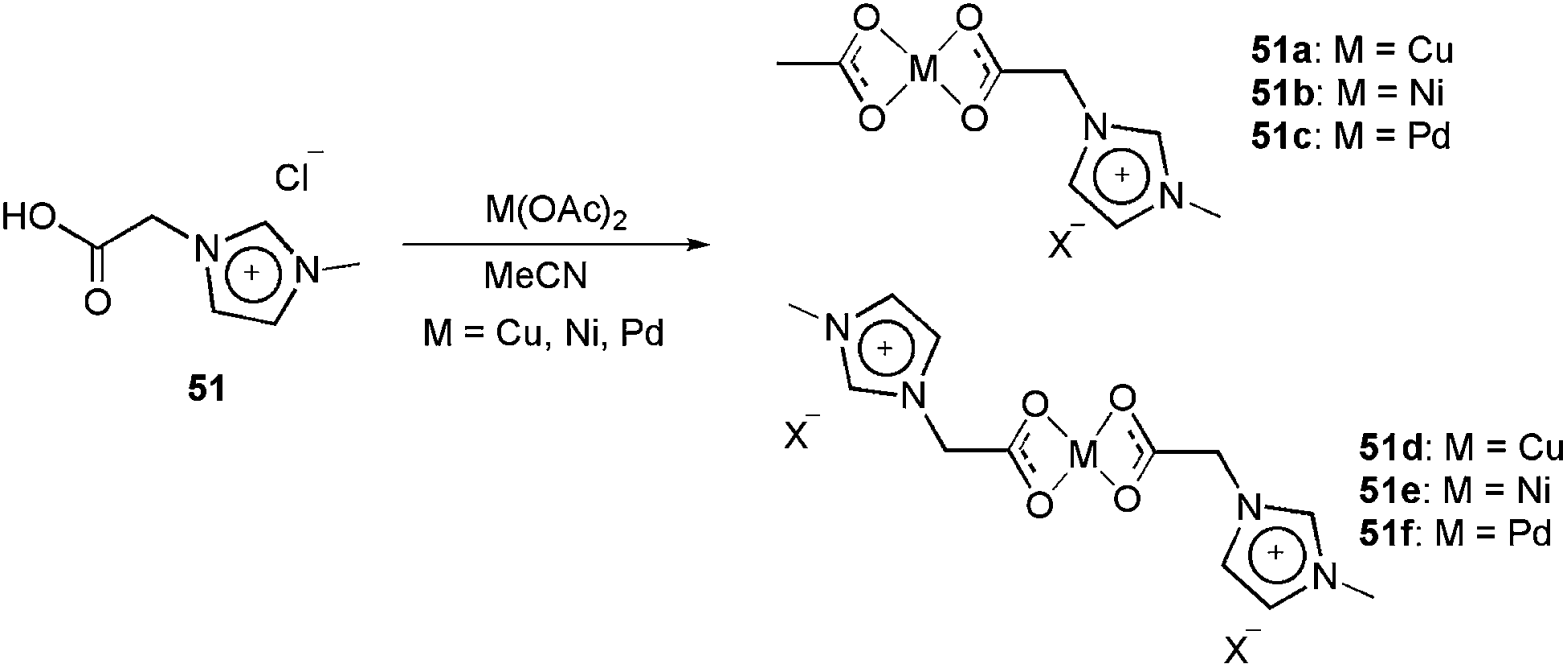
Reaction of the charge-tagged carboxylate ligand **51** with Cu(OAc)_2_, Ni(Oac)_2_ and Pd(OAc)_2_, generating the corresponding mono- and dicationic charge-tagged metal complexes.

The charge-tagged carboxylate ligand **51** was synthesised by Neto and co-workers.^17^ This ligand was reacted with Cu(OAc)_2_, Ni(OAc)_2_ and Pd(OAc)_2_, generating the corresponding mono- and dicationic charged-tagged metal complexes **51a–51f** (Scheme 26).

The ion-tagged complexes were characterised by ESI(+)-MS and ESI(+)-MS/MS. For both the Cu and Ni complexes, the formation of metallic NHC carbenes was observed in the mass spectra. The palladium dicationic carboxylate was successfully used in the Heck and Suzuki reactions. In both reactions, the activity of **51f** was greater than that of Pd(OAc)_2_. Although the palladium carbene was not observed in the ESI-MS experiments, the superior activity of the CTL was attributed to formation of this type of intermediate.

It must be mentioned that the same research group described the detection of this NHC carbene through negative-ion ESI-MS [ESI(–)-MS] experiments of COOH- and SO_3_H-substituted IL. In the gas phase, the doubly deprotonated (imidazolium and COOH or SO_3_H) NHC carbene was detected. Moreover, the behaviour of these carbenes in the gas phase was similar to those in the liquid phase, because they can react with CO_2_ and generate organic carboxylates at the C2 positions of imidazolium.^101,102^

**Scheme 27:**
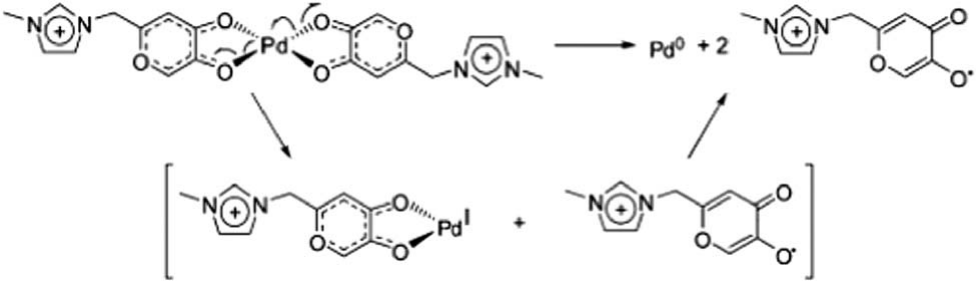
Proposed Pd-reduction by ligand decomposition.

Key reaction intermediates associated with Pd catalysis could also be detected and characterized due to the presence of the charge tag on the Pd-complex (Scheme 27).^103^ The new charge-tagged Pd-complex is also shown to function as an active catalyst “on water” with the advantage of using cheaper and less reactive aryl chloride substrates in a phosphine-free version of the Heck reaction.

**Scheme 28:**
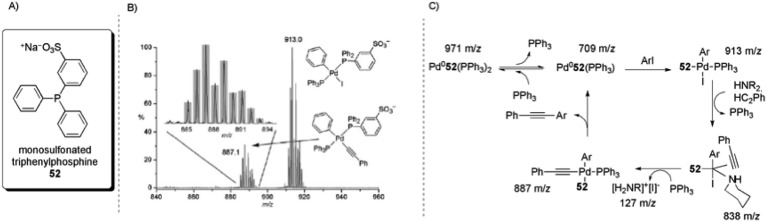
(A) Structure of monosulfonated triphenylphosphine (**52**). (B) ESI(–)-MS of Pd(PPh_3_)_4_ + [PPN][**52** ^–^] + 100 × (PhI + PhC_2_H + NEt_3_) in CH_2_Cl_2_. Inset: isotope pattern match for [Pd(**52** ^–^)(PPh_3_)(Ph)(C_2_Ph)]. (C) Intermediates that were directly observed by ESI(–)-MS and the proposed catalytic cycle. Reproduced from ref. 104 with permission from the Royal Society of Chemistry.

The use of tagged substrates^19^ and ligands^104^ has been described for the study of the intermediates of the palladium-catalysed Sonogashira cross-coupling reaction through ESI(+)-MS and ESI(–)-MS experiments. The commercial salt of monosulfonated triphenylphosphine [Na]^+^[PPh_2_(*m*-C_6_H_4_SO_3_)]^–^ ([Na]^+^[**52**]^–^) was used as a precursor to obtain CH_2_Cl_2_-soluble [(Ph_3_P)_2_N]^+^[PPh_2_(*m*-C_6_H_4_SO_3_)]^–^ ([PPN]^+^[**52**]^–^). An equimolar amount of the CTL [PPN][**52**] was added to a Pd(PPh_3_)_4_ solution. This mixture provided an ESI(–)-MS spectrum that consisted of only [Pd(**52** ^–^)PPh_3_] and [Pd(**52** ^–^)(PPh_3_)_2_]. After PhI was added, all of the Pd(0) species were consumed and the oxidative addition product [Pd(**52** ^–^)(PPh_3_)(Ph)(I)] could be detected. Upon the addition of phenylacetylene and NEt_3_, the intermediate [Pd(**52** ^–^)(PPh_3_)(Ph)(C_2_Ph)] was formed (Scheme 28A). Additionally, by using piperidine, the intermediate [Pd(**52** ^–^)(HNR_2_)(Ph)(I)(HC_2_Ph)] (*m*/*z* = 838; Scheme 28B) could be identified. Collision-induced dissociation (CID) experiments were performed on this complex and the loss of phenylacetylene and then piperidine was observed. Moreover, there was no detected loss of benzene, indicating that the triple bond is π-coordinated in this complex. These results led to the catalytic cycle proposed in Scheme 28C.

#### Olefin hydrogenation

**Scheme 29:**
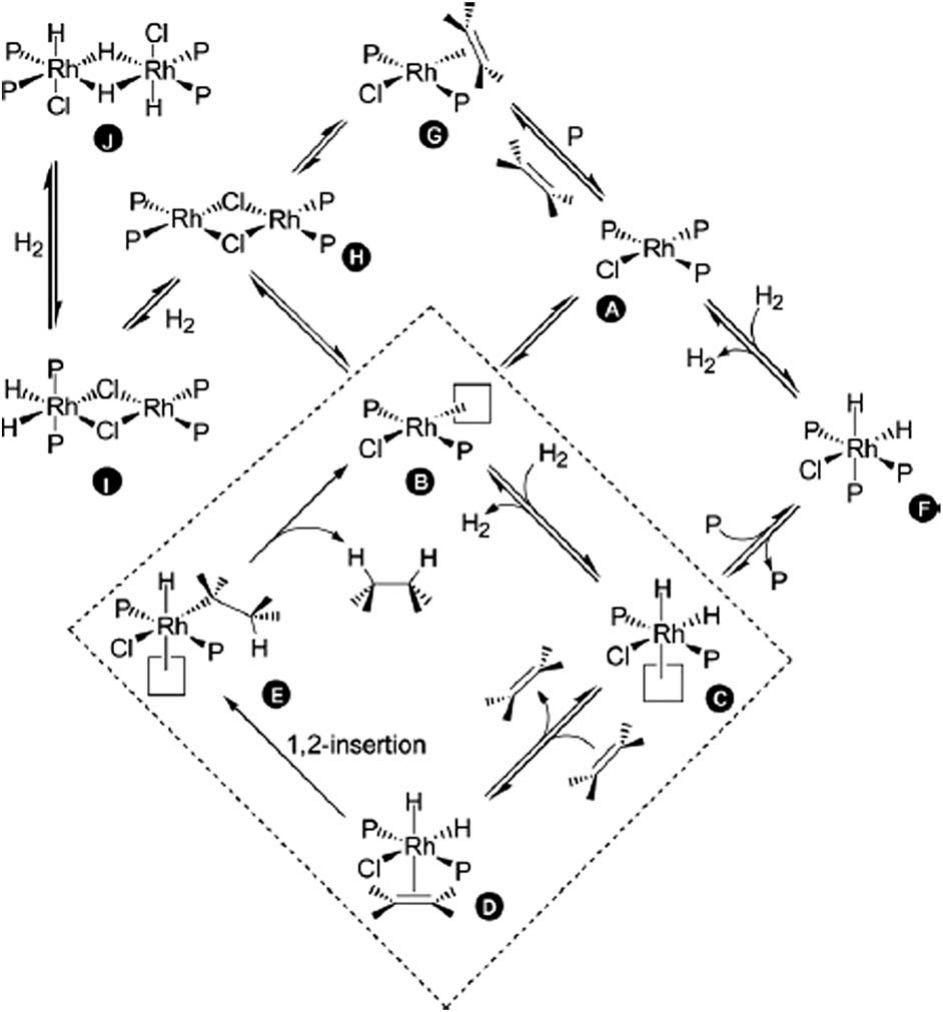
Catalytic cycle of the RhCl(PPh_3_)_3_-catalysed hydrogenation of olefins. Reproduced from ref. 109 with permission from the Royal Society of Chemistry.

Scheme 29 has been studied in detail,^105–108^ and both the in-cycle (**B–E**) and off-cycle (**A**, **F–J**) species have been characterised. For this reason, the reaction can be used to evaluate the reliability of ESI-MS for detecting intermediates during catalysis. However, because all of the species are neutral, it is necessary to charge-tag them.

Therefore, Ph_2_P(CH_2_)_*n*_PPh_2_ (*n* = 1, 2, 4, 6) diphosphines were selectively monobenzylated to produce charge-tagged phosphonium/phosphine [Ph_2_P(CH_2_)_*n*_PPh_2_CH_2_Ph]^+^ ligands. These CTLs were used to dope RhCl(PPh_3_)_3_ under hydrogenation conditions in order to detect the reaction intermediates in ESI(+)-MS.^109^

Ligand **53** (*n* = 4) was added to a solution of RhCl(PPh_3_)_3_ in chlorobenzene. The reaction was analysed before H_2_ was added, after H_2_ was added and after the addition of cyclohexene. The results are summarised in Table 1. Initially, various intermediates, in which either one or two PPh_3_ ligands had been replaced by **53**, were detected (for instance [RhCl(PPh_3_)(**53** ^+^)_2_] and [RhCl(PPh_3_)_2_(**53** ^+^)]). The reactive **B**-type (see the catalytic cycle) species [RhCl(PPh_3_)(**53** ^+^)] and [RhCl(**53** ^+^)_2_]_2_ were also detected; however, by varying the cone voltage it could be demonstrated that these species were formed by the fragmentation of the **A**-type species. When hydrogen was bubbled through the solution, an immediate change in the mass spectrum was observed. Even at a low cone voltage, a significant amount of the three-coordinate species [RhCl(PPh_3_)(**53** ^+^)] appeared. In addition, the hydrogenated species [RhCl(PPh_3_)(**53** ^+^)_2_H_2_], [RhCl(PPh_3_)_2_(**53** ^+^)H_2_], [RhCl(PPh_3_)(**53** ^+^)_2_H_2_BF_4_], [Rh_2_Cl_2_(PPh_3_)_3_(**53** ^+^)H_2_] and [Rh_2_Cl_2_(PPh_3_)_3_(**53** ^+^)H_4_] could be detected. After the addition of cyclohexene, despite the change in speciation, no alkene-coordinated (**D**-type) species were detected. However, bubbling ethylene through the hydrogen-saturated solution resulted in the appearance of a small amount (*ca.* 2%) of [RhCl(PPh_3_)(**53** ^+^)(H_2_C=CH_2_)] (**G**-type). In summary, by using this method, all the off-cycle (**A**, **F–I**) species could be identified rapidly.

**Table 1:** Species detected in ESI(+)-MS for the reaction of RhCl(PPh_3_)_3_ with **53**. Adapted from ref. 109

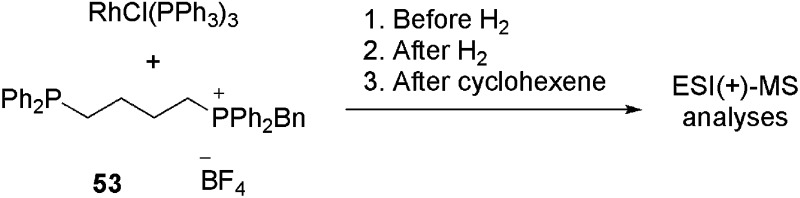						
Species	Cycle	Identity	*m*/*z*	Intensity before H_2_ addition	Intensity after H_2_ addition	Intensity after cyclohexene addition
Free ligand		**53** ^+^	517.2	13	13	4
RhClP_2_	B	[RhCl(**53** ^+^)_2_]_2_	586.2	11	3	<1
	B	[RhCl(PPh_3_)(**53** ^+^)]	917.2	24	100	6
RhP_3_^+^		[Rh(PPh_3_)_3_]^+^	889.2	15	8	30
RhClP_3_	A	[RhCl(PPh_3_)(**53** ^+^)_2_]	717.2	16	9	<1
	F	[RhCl(PPh_3_)(**53** ^+^)_2_H_2_]	718.2	—	3	<1
	A	[RhCl(PPh_3_)_2_(**53** ^+^)]	1179.3	100	10	100
	F	[RhCl(PPh_3_)_2_(**53** ^+^)H_2_]	1181.3	—	77	<1
	A	[RhCl(PPh_3_)(**53** ^+^)_2_ BF_4_]	1521.4	4	<1	2
	F	[RhCl(PPh_3_)(**53** ^+^)_2_H_2_ BF_4_]	1523.4	—	2	<1
Rh_2_Cl_2_P_4_	H	[Rh_2_Cl_2_(PPh_3_)_3_(**53** ^+^)]	1581.2	6	2	3
	I	[Rh_2_Cl_2_(PPh_3_)_3_(**53** ^+^)H_2_]	1583.2	—	2	<1
	J	[Rh_2_Cl_2_(PPh_3_)_3_(**53** ^+^)H_4_]	1585.2	—	<1	<1

**Scheme 30:**
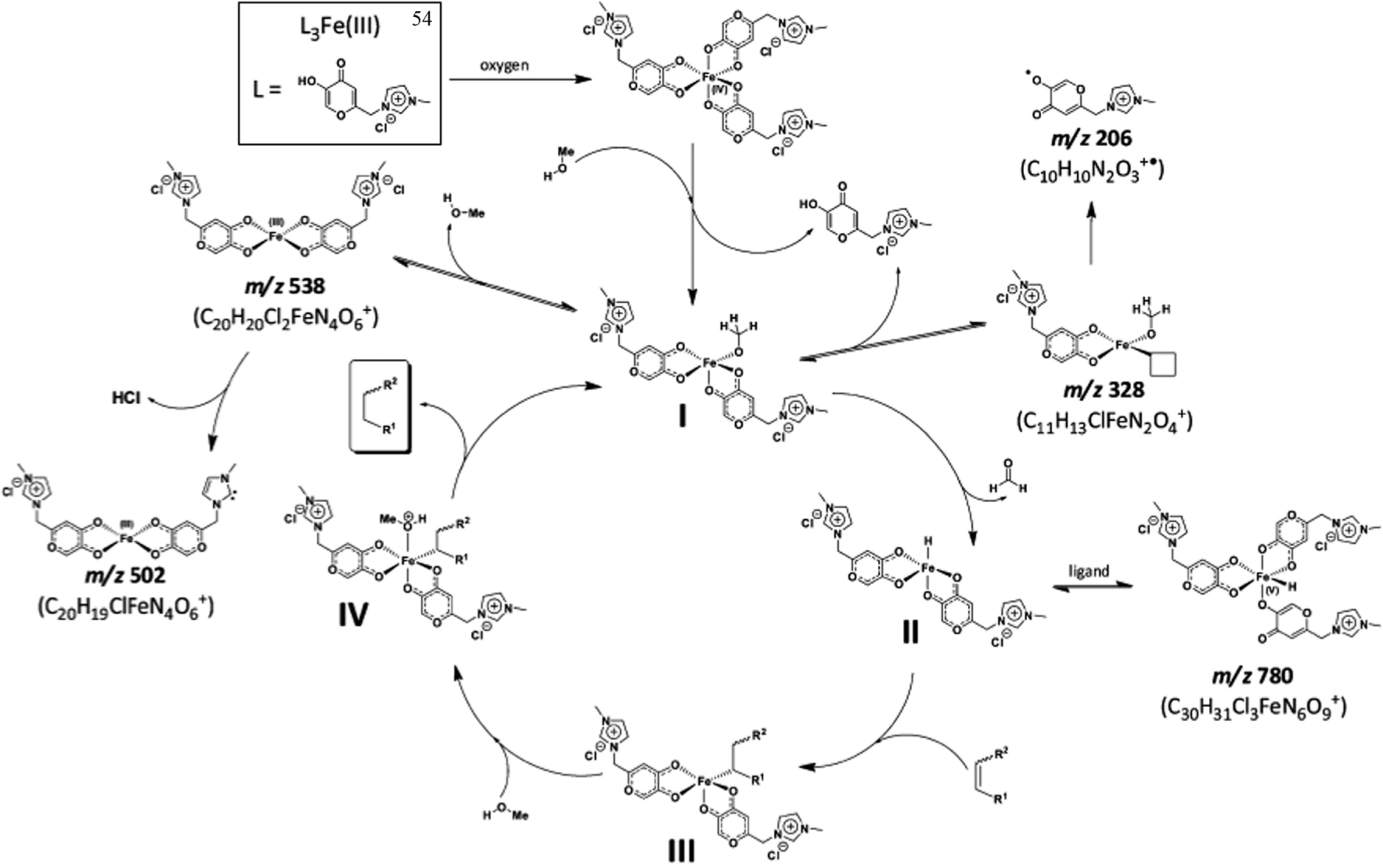
Proposed catalytic cycle for olefin reduction catalysed by **54** in the presence of methanol. Major ions detected in the ESI(+)-QTOF-MS experiments are indicated by their *m*/*z* values, and proposals for the origins of the intermediates are shown. Reprinted with permission from ref. 112. Copyright (2012) American Chemical Society.

An iron(iii) complex with three ionic tags, **54**, was applied to the reduction of olefins in imidazolium-based ILs under oxidative conditions.^110^ The catalyst was very efficient in promoting reactions involving biomass derivatives. ICP analysis revealed that the use of this catalyst greatly prevented catalyst leaching, and as little as 2 ppm of Fe was detected in the organic phase of the reaction. This indicates the efficient anchorage of the complex in the BMI·NTf_2_ IL. Moreover, the activity of this complex remained unchanged after ten runs. Some important mechanistic insights for this new reaction were also provided, mostly based on ESI quadrupole time-of-flight MS (ESI-QTOF-MS), as can be seen in Scheme 30. Based on these experiments, a catalytic cycle that includes a high-valence Fe atom was proposed.

#### Metal-catalysed multicomponent Biginelli reactions

The Biginelli reaction^111^ is an acid-catalyzed, three-component reaction between an aldehyde, a β-ketoester and urea that allows the rapid and facile synthesis of dihydropyrimidones, which are interesting compounds with potential for pharmaceutical applications.

**Fig. 5:**
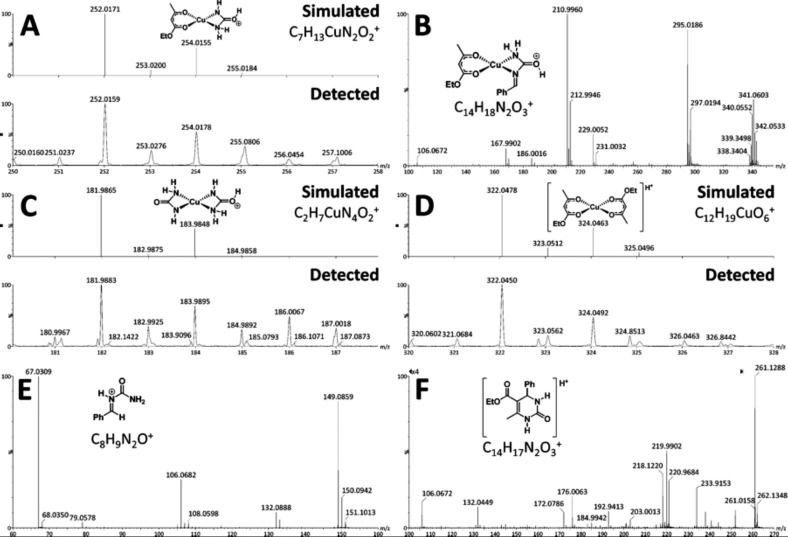
Key intermediates and products detected (and characterised) by ESI(+)-MS: (A) C_7_H_13_CuN_2_O_2_^+^; (B) C_14_H_18_N_2_O_3_^+^; (C) C_2_H_7_CuN_4_O_2_^+^; (D) C_12_H_19_CuO_6_^+^; (E) iminium intermediate C_8_H_9_N_2_O^+^; and (F) C_14_H_17_N_2_O_3_^+^. Reprinted with permission from ref. 112. Copyright (2012) American Chemical Society.

This reaction catalysed by a Lewis acid in an IL was evaluated by Neto and co-workers using ESI-MS.^112^ This multi-component reaction was used to obtain the biologically active 3,4-dihydropyrimidin-2(1H)-one (DHPMs). As indicated by ESI-MS experiments, the ionic medium plays a fundamental role in this synthesis, owing to the stabilisation of the charged intermediates. Presently, there are three major pathways accepted for this reaction: (i) the iminium, (ii) the Knoevenagel and (iii) the enamine mechanisms.^113–115^ Through this work, the authors could detect some key intermediates and products of the reaction by using ESI(+)-MS(/MS) (Fig. 5). The online monitoring of the Biginelli reaction allowed the authors to detect and characterise the so-called iminium intermediate (Fig. 5E), indicating that, under these conditions, the preferred mechanistic pathway is the iminium mechanism. No key intermediate of the enamine mechanism could be detected.

**Fig. 6:**
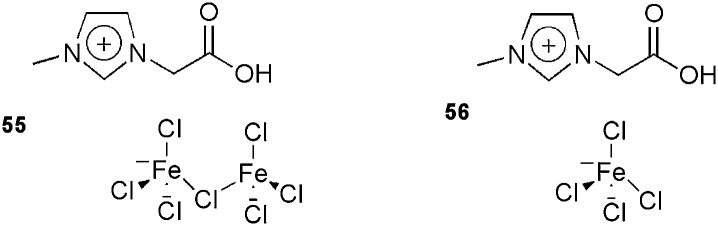
Ion-tagged iron catalysts used in the Biginelli reaction.

This reaction has also been studied using the new ion-tagged iron catalysts **55** and **56** (Fig. 6) in ILs.^116^ High yields were obtained and the system could be recovered and reused up to eight times. High-resolution ESI-QTOF-MS experiments were performed to determine the reaction mechanism.

The three proposed mechanisms for the Biginelli reaction were investigated by ESI-QTOF-MS and MS/MS. Only the intermediates from the iminium mechanism were detected. No intermediates from the Knoevenagel or enamine pathways were found.

#### Iron-catalysed olefin epoxidation

The ionically tagged imidazolium-based iron(iii) complex **54** was used in the oxidation of alkenes in imidazolium-based ILs in air (also hydrogen peroxide).^48^ The epoxidised olefin was obtained in very good yields (84–91%) and at least ten recycling reactions could be performed. ESI-QTOF-MS experiments were performed to achieve a better understanding of the active catalytic species that promoted epoxidation. The results pointed firmly towards two different oxidation states of the metal centre [Fe(iv) and Fe(v)], depending mostly on the selection of the oxidising agent. Hydrogen peroxide oxidation may proceed through a radical pathway and may use a Fe(iv) species in its catalytically active form, whereas air oxidation may favour a concerted mechanism with an Fe(v)-based intermediate as the active species to promote epoxidation. Some important mechanistic insights could be provided based on the ESI-QTOF-MS results for the oxidation reaction, which clearly indicated that oxidation can take place by two different pathways, depending on the reaction conditions, that is, a radical or a concerted mechanism.

## Conclusions and perspectives

It is evident that, with the advent of IL-phase chemistry, a new class of organic charged ligands has emerged. Contrary to classical water-phase catalysis, for which negatively charged ligands (in most cases, P-containing ligands) are used, IL-phase catalysis is mainly based on positively charged ligands. This is necessary, because an incorrect solvent pair can lead to low conversions, poor product extraction, no phase separation and catalyst lixiviation. Using this approach, various classical homogeneous catalytic processes have been successfully transposed to liquid–liquid biphasic catalysis. However, so far, it is only in rare cases that the changes to the mechanism and selectivity of the transformations, imposed by the intrinsic ionic nature of the media, have been addressed. Moreover, even fewer insights have been gathered for the supramolecular organisation imposed by these highly organised fluids on the catalytically active sites. Therefore, a more detailed investigation on the mechanistic pathway in ionic media is necessary to allow the design of more efficient catalysts (for improved conversion, selectivity and sustainable catalytic processes). Notably, CTLs have also been used with a high degree of success in colloidal catalytic process.

It also appears that the use of CTLs has opened a new window of opportunities for the investigation of reaction pathways through ESI-MS. This approach has already proven to be one of the most important tools for detecting several species involved in various catalytic reactions directly from the solution. It is also expected that, in view of the virtually non-volatile nature of ILs, other techniques will be used to probe and monitor *in situ* reactions performed in solution, such as techniques requiring a high vacuum and those that are currently limited to solid-state chemistry, for example, X-ray photoelectron spectroscopy, low-energy ion scattering spectroscopy and TEM. This may also lead to important *in situ* solution-phase information that can be used for the design of new and more efficient catalysts.
